# Rat-Bite Fever due to the genus *Streptobacillus*

**DOI:** 10.3934/microbiol.2024040

**Published:** 2024-11-05

**Authors:** Mehdi Fatahi-Bafghi

**Affiliations:** 1 Infectious Diseases Research Center, Shahid Sadoughi Hospital, Shahid Sadoughi University of Medical Sciences, Yazd, Iran; 2 Department of Microbiology, Faculty of Medicine, Shahid Sadoughi University of Medical Sciences, Yazd, Iran

**Keywords:** *Streptobacillus*, rat-bite fever, rodent, treatment, zoonotic infection, PCR, Haverhill fever

## Abstract

Rat-bite fever (RBF) is a zoonotic infection and systemic febrile illness transmitted to humans by *Rattus* spp. contacts following a scratch, bite, or touching excrement, such as urine, feces, and oral secretions. Infection with members of the genus *Streptobacillus* is the most common cause of this infectious disease. In this review article, we updated the knowledge on the RBF caused by the genus *Streptobacillus* based on the isolation and identification methods, virulence factors, clinical signs, differential diagnoses, antibiogram, treatment, geographical distribution, and epidemiology. Moreover, the present paper's comprehensive analysis of over 200 infection cases attributed to this genus, spanning from 1915 to 2023, sheds light on its epidemiology and provides valuable insights for the future.

## 1. Introduction

Rodents are common sources of diseases in humans. Some other zoonotic diseases include salmonellosis, leptospirosis, hantavirus infections, Lassa fever, zoonotic babesiosis, lymphocytic choriomeningitis, taeniasis-like *Capillaria* spp., and plague [1]–[3]. More than 2,300 years ago, Wagabhatt described cutaneous wounds caused by rat bites in India, and many researchers believe that rat bite fever (RBF) was first reported in this country [4]. Some bacteria, such as *Corynebacterium* spp., *Fusobacterium* spp., *Leptospira* spp., *Staphylococcus* spp., *Pasteurella* spp., *Streptobacillus* spp., and *Spirillum minus* (the name of the bacterium is not included in the approved list due to lack of a type strain), have been isolated from human at the site of the lesion following rat bites [4]. In 1914, Hugo Schottmüller isolated one bacterium from a blood culture in a rat bite patient and named it *Streptothrix muris ratti*. Levaditi, Nicolau, and Poineloux changed the name of the organism to *Streptobacillus moniliformis* in 1925 [5]. *Streptobacillus* are a Gram-negative rod, facultative anaerobic, non-capsulate, alpha (α) or non-hemolytic, and non-motile species that cause various infections in humans and animals [6]–[14]. *S. moniliformis* and *Streptobacillus notomytis* are the causes of RBF [11],[15],[16]. In 2021, *Streptobacillus felis* was reported using the molecular method (sequencing of the 16S rRNA and *gyrB* genes) from the skin lesion of an 18-year-old man from Germany [17]. *S*. *moniliformis* is a zoonotic bacterium that is transmitted through rat bites and scratches [15],[18],[19], licks [20], or following the handling of deceased rats [18],[19]. *Rattus* are typically contaminated with *S*. *moniliformis* in the range of 50–100%, while pet rats have contamination rates ranging from 10–100% [20]. Three studies by Julius et al., Azimi et al., and Azimi et al. reported the prevalence of contamination with *S. moniliformis* in *Rattus* spp., 50.9%, 30%, and 23% from South Africa and Iran in 2012, 2021, and 2021, respectively [21]–[23]. Moreover, de Cock et al. collected 412 samples of wild rats, and 2% of them were contaminated with *S*. *moniliformis* [24]. These rats often carry the bacterium asymptomatically in the nasopharyngeal and oropharyngeal tracts, larynx, and mouth and excrete the organism through urine, feces, and eye and oral secretions such as saliva or mucosa [20],[25]–[32]. Other rodents, such as laboratory rats and mice [33]–[37], are also susceptible to this infection. Also, this organism has been isolated from turkeys [15],[38]. The most common clinical signs of RBF infections in humans include fever, maculopapular, and petechial or pustular rash [15]. This organism can act as a secondary invader [39] together with probable pathogens such as *Mycoplasma pulmonis* (renamed to *Mycoplasmopsis pulmonis* [40]) and *Rodentibacter pneumotropicus* causing infection in the middle ear (Otitis media) [35],[41], bronchopneumonia [42], chronic pneumonia [43], and conjunctivitis [44]. Diagnosis of RBF caused by the genus *Streptobacillus* is a diagnostic dilemma [45] for four reasons: lack of attention to the bite or contact with rodents, non-specific clinical signs, fastidious growth characteristics, and lack of reliable diagnostic methods for non-notifiable and non-communicable diseases with broad antimicrobial susceptibility [46],[47]. Another manifestation of RBF is known as “sodoku” derived from the Japanese words “sō” (meaning rat) and “doku” (meaning poison). This variant is associated with the bacterium *Spirillum minus*, although it remains beyond the scope of this review. The current review aims to update the knowledge on the RBF due to the genus *Streptobacillus* based on the isolation and identification methods, virulence factors, clinical signs, differential diagnoses, antibiogram, treatment, geographical distribution, and epidemiology.

## 2. Nomenclature of the genus *Streptobacillus*

In the Leptotrichiaceae family, there are eight genera, including *Caviibacter* [48], *Leptotrichia* [49], *Oceanivirga* [50], *Pseudoleptotrichia* [51], *Pseudostreptobacillus* [52], *Sebaldella* [53], *Sneathia* [54], and *Streptobacillus* [5]. The genus *Streptobacillus* includes five species described as follows: *Streptobacillus canis* (isolated from a dog) [9], *Streptobacillus felis* (isolated from a cat with pneumonia) [8], *S. moniliformis* (isolated from a blood culture by Hugo Schottmüller) [5], *S*. *notomytis* (first isolated from a spinifex hopping mouse with septicemia in Australia and black rats in Japan) [10], and *Streptobacillus ratti* (isolated from a black rat by Eisenberg et al. in 2016) [7]. *Streptobacillus hongkongensis* was first introduced by Woo et al. in 2014 when isolated from patients with septic arthritis [55] and this species was reclassified into a novel genus as *Pseudostreptobacillus* by Eisenberg et al. in 2020 [52]. Also, *Streptobacillus actinoides* was isolated from the pneumonic lungs of calves [56]; however, the nomenclatural status of this bacterium has not been validly published [57].

## 3. Isolation methods and cultural properties

Isolation and culture of *Streptobacillus* is the gold standard for streptobacillosis diagnosis. Five species in the genus *Streptobacillus* are fastidious, need enriched culture media and specific incubation conditions, and are slow-growing [32]. The incubation period for *S. ratti*, *S. canis*, *S. moniliformis*, and *S. felis* on Columbia blood agar supplemented with 5% sheep blood with the capnophilic condition of 5–10% CO_2_ (the use of a candle jar or humidified CO2 incubator) is between 1–3 days (up to 7 days), and the optimum temperature has been reported 35–37 °C [7]–[9],[32],[58],[59]. The colonial morphology of these bacteria is small (1–2 mm in diameter), smooth, shiny, and nonhemolytic (strains such as *S. felis* 131000547^T^ and *S. ratti* OGS16^T^ showed a hemolysis phenotype on sheep blood agar plates [7],[8],[59]–[61]). Tryptone soy agar supplemented with 20% horse serum, Schaedler agar, peptone broth, and brain heart infusion supplemented with 20% cattle or horse serum are the best media for the cultivation of *S. ratti*, *S. canis*, *S. notomytis*, *S. moniliformis*, and *S. felis*. However, these species cannot grow on the Gassner, MacConkey agar [7]–[9],[10],[62],[63], and Löwenstein-Jensen medium [64],[65]. Also, Dendle et al. reported that *S. moniliformis* could not grow on the nutrient agar or chocolate agar [66]. *S. felis* could grow on the media at 20–43 °C but could not grow at 10 or 50 °C [8]. Eisenberg et al. reported that *S. canis* could grow at 20–43 °C, but growth was not achieved at 6, 16, 20, 43, 44, or 50 °C [9]. In another study, it was reported that the growth of *S. notomytis* on the Columbia agar with 5% sheep blood was weakly at 43 °C, but this bacterium was not grown at 10, 20, or 50 °C [10]. The incubation period for this bacterium is 2–5 days at 37 °C under a capnophilic condition of 10% CO_2_ on Columbia agar with 5% sheep blood [10]. Selective medium agar containing nalidixic acid, colistin, or trimethoprim/sulfamethoxazole is suitable for primary isolation of *Streptobacillus* species from sites with microbiota such as mucosal sites [25],[38],[67]. Nevertheless, Uddin et al. reported that they could not observe the growth of this bacterium on Columbia colistin-nalidixic acid agar [63]. Lopez et al. isolated *S. moniliformis* with culture onto Blood agar, Chocolate agar, and Thioglycolate broth under incubation at 35 °C with supplementing atmospheric CO_2_ for two days from joint fluid [68]. Rosen and Denzer recovered *S. moniliformis* with culture onto tryptose broth with human serum, tryptose broth with ascitic fluid, tryptose broth with rabbit's blood, and brain-liver-heart semisolid media under capnophilic condition [69]. Other studies have shown that *S. moniliformis* can grow well on Loeffler's medium with or without blood serum [70]–[73], ascitic agar, and ascitic bouillon after in 24 hours at 37 °C [70]. Dendle et al. isolated *S. moniliformis* from joint fluid in cooked meat broth under CO_2_ at 35 °C for 72 hr [66]. Hagelskjaer et al. reported the isolation of *S. moniliformis* from pus using horse blood agar with 48 h incubation at 35 °C under 5% CO_2_ [74]. Rupp et al. recovered *S. moniliformis* from blood culture with a biphasic system (This media contains a trypticase soy agar slant and trypticase soy broth) [75]. Dijkmans et al. emphasized that sampling the lesion in the brain abscess is necessary for bacterial identification [76]. Susceptibility to anticoagulants such as sodium polyanethol sulfonate (SPS) used in standard aerobic blood culture media for blood specimens of patients suspected of bacteremia or septicemia, may inhibit the growth of *S. moniliformis*. Thus, it decreases the sensibility of blood culture [20],[62],[75],[77] (Other bacteria such as *Capnocytophaga* spp., *Gardnerella vaginalis*, *Neisseria meningitidis*, *Mycoplasma hominis*, and *Peptostreptococcus anaerobius* are susceptible to SPS with variable concentrations [65]). Growth of this bacterium is inhibited at concentrations of 0.0125% of SPS, while standard aerobic blood culture bottles contain 0.05% SPS [62],[78]. Nevertheless, *S. moniliformis* was successfully isolated from blood culture with the Bactec system that used polyanethole sulphonate as an anticoagulant [79]–[81]. Also, some blood culture anaerobic media do not contain SPS, which has been used to isolate *S. moniliformis* [82]. It is necessary to use other anticoagulants in blood culture for the isolation *S. moniliformis* [82]. The sensitivity of other species to SPS has not been proven. The volume of 8–10 mL of blood with anticoagulant as with sodium citrate as the anticoagulant (10 mL sodium citrate, 2.5%) will improve the isolation of the bacterium [80],[83]. Loridant et al. proposed using a shell vial cell culture to isolate *S. moniliformis* from blood culture media suspected that the bacteria were dead [65]. Fukushima et al. isolated *S. notomytis* from the pustule sample with ATCC medium 488 broth containing heart infusion broth containing 0.9% peptone, 18.2% horse serum, and 0.045% glucose under anaerobic conditions at 37 °C [16]. Another study by Kusuda et al. isolated *S. notomytis* from blood culture with the Bactec system [84]. In cases with clinical signs following a rodent bite, blood and bite wound culture should be done before starting antibiotic therapy [74]. Isolating of *S. moniliformis* and *S. notomytis* from blood cultures is not reliable enough as a diagnostic method in bacteremia, and researchers have reported rates of culture failure as high as 33% [85]. *S. moniliformis* isolated from other clinical specimens include pus material, amniotic fluid, material obtained by puncture (parasagittal burr), synovial fluid, cerebrospinal fluid (CSF), articular fluid, ovarian abscess and intrauterine device, purulent material extracted from nodule, seropurulent material, pustular material, skin biopsy, bone, wrist bulla, spinal disc and peripheral joint specimens, empyema intraoperative sample, and aspiration of the L2-L3 disc (Table S1). Also, *S. notomytis* has been isolated from joint fluid, blood, and skin pustule samples (Table S2).

## 4. Phenotypic identification

Simultaneous application of molecular methods is necessary for accurate identification at the genus and species level. Gram staining showed that *S. ratti*, *S. canis*, *S. notomytis*, *S. moniliformis*, and *S. felis* are Gram-negative fusiform to filamentous or pleomorphic, without spore and capsule production [7]–[10],[20]. Also, these bacteria are negative for acid-fast staining [7]–[10],[64],[68] (positive in bacteria that have mycolic acid in their cell wall structure [86]–[88]). At times, the morphology of *S. moniliformis* unveils lateral bulbar swellings, presenting irregular arrangements akin to chains and clumps [7]–[10],[12]. The diameter of colonies in *S. moniliformis* is ranging from 1–2 mm [89]. In 1995, Bottone et al. reported Gram variable bacillary and pleomorphic forms with bulbous swellings identified as nutritionally deficient streptococci [90]. The morphology of *Streptobacillus* species appears as bread-crumb growth like floccules or puff-balls in liquid cultures medium (ex. Thioglycolate broth) after 2–7 days [15],[20],[68]. In the genus *Streptobacillus*, *S. moniliformis* has two variant types: the L-form and the bacillary form [15],[59]. Distinguishing L-phase variants from parent strains is as follows: difference in the colonial morphology (morphology of the colony is similar to fried-egg appearance and their distinction from colonies of *Mycoplasma* is difficult), certain physical properties, and significantly high-level penicillin resistance [91]. For differentiation of *Streptobacillus* from other genera, such as *Cardiobacterium*, *Actinobacillus*, and *Haemophilus*, that live in the same habitat, phenotypic tests include the need for serum supplement for growth in liquid media, microscopic characteristics, enzyme activity which are negative for catalase, nitrate reductase, and oxidase, and failure to produce indole from tryptophan, are suitable for identification at the genus level [92],[93]. Eisenberg et al. reported that phenol red solution as an indicator base supplemented with carbohydrates is suitable for biochemical tests. Also, biochemical tests should be incubated for seven days at 37 °C before reading [47]. *S. moniliformis* produced acid without gas from galactose, dextrin, trehalose, glucose, maltose, fructose, and sucrose but is negative for adonitol, sorbitol, rhamnose, raffinose, inulin, inositol, erythritol, dulcitol, arabinose, mannitol, mannose, lactose, salicin, and xylose [14],[62],[68],[80],[94],[95]. Lambe et al. reported that some negative biochemical tests for *S. moniliformis* include urease, gelatinase, lysine decarboxylase, ornithine decarboxylase, and utilization of citrate. Also, this bacterium's pigment production is negative on fluorescent agar [62]. Another study reported that *S. moniliformis* produced acid from glucose, salicin, and occasionally from maltose and lactose [96]. Cohen and colleagues reported that acid production for identifying *S. moniliformis* is negative for fructose or trehalose but positive for galactose, glucose, maltose, and salicin [97]. Stuart-Harris et al. also reported that this bacterium produced acid from glucose, lactose, maltose, raffinose, salicin, and sucrose; however, they reported no acid from dulcitol, inulin, and mannitol [98]. Sens et al. reported that this organism produced acid from levulose, maltose, glucose, and mannose. Additionally, it hydrolyzed starch [99]. Frans et al. reported one isolate of *S. moniliformis* that can produce acid from glucose, galactose, levulose, maltose, mannose, and salicin [100]. Pins et al. isolated three strains of *S. moniliformis* from ovarian abscess and purulent material extracted from nodule that they failed in acid production from mannitol, maltose, lactose, glucose, sucrose, and xylose [89]. Edwards et al. reported three isolates of *S*. *moniliformis* that produced acid from salicin [95]. The diversity in fermentation patterns observed across different studies can likely be attributed to four main factors: differences in the carbohydrate bases utilized, variations in the percentage of carbohydrate utilization, genetic diversity among strains, and variations in the incubation time for carbohydrates [62]. To date, matrix-assisted laser desorption/ionization-time of flight mass spectrometry (MALDI-TOF MS) is increasing in microbiology laboratories due to the high accuracy in differentiation and identification of bacteria at the genus and species level [101]. MALDI-TOF MS needs fresh pure culture and appropriate database entries for all *Streptobacillus* species [67]. Eisenberg et al. used MALDI-TOF MS for the identification of *S*. *ratti*, *S. canis*, *S. notomytis*, *S. felis*, and they proved it is a suitable tool for differentiating all validly published names of *Streptobacillus* species [7]–[10]. Other researchers have also identified *S. moniliformis* by MALDI-TOF MS [63],[85],[102]. Nevertheless, Fokkema et al. reported that they could not identify their bacterium successfully with this method [103] and that the databases of their analyzers were presumably incomplete. Transmission electron micrographs have been done for all species but could not distinguish species in this genus [47]. Phenotypic tests for *Streptobacillus* species identification are shown in Table 1.

**Table 1.** Some of the phenotypic characterizations of *Streptobacillus* spp.

Characteristic	Species				
	*Streptobacillus moniliformis* DSM 12112^T^ [7],[8]	*Streptobacillus ratti* DSM 101843^T^ [7]	*Streptobacillus felis* 131000547^T^ [8]	*Streptobacillus notomytis* AHL 370-1^T^ [10]	*Streptobacillus canis* DSM 110501^T^ [9]
Hemolysis on SBA	-	+	+	-	+
Indole production	-	-	-	-	-
Alkaline phosphatase	W	-	+	-	+
Phosphatase	-	-	-	-	-
Esterase (C4)	W	-	+	+	+
Esterase lipase (C8)	+	+	+	+	+
Neuraminidase	+	ND	-	+	ND
Leucine arylamidase	-	-	-	W	+
a-Chymotrypsin	+	+	-	+	+
Phenylalanine arylamidase	+	+	-	+	+
Acid phosphatase	W	-	+	-	+
Naphthol-AS-BIphosphohydrolase	-	-	-	-	w
Cytochrome oxidase	-	-	-	-	-
Catalase	-	-	-	-	-
Nitrate reduction	-	-	-	-	-
Proline aminopeptidase	+	ND	+	+	ND
Hydroxyproline aminopeptidase	+	ND	+	+	ND
Arginine aminopeptidase	+	ND	+	+	ND
Chitinase	-	ND	+	-	-

*S. moniliformis* is urease-negative; +, positive; -, negative; w, weak; ND, not determined; α-Glucosidase, β-Glucuronidase, naphthol-AS-BI-phosphohydrolase, cystine arylamidase, valine arylamidase, and aspartyl aminopeptidase were negative for *S. moniliformis* [60].

## 5. Molecular identification

**Figure 1.**
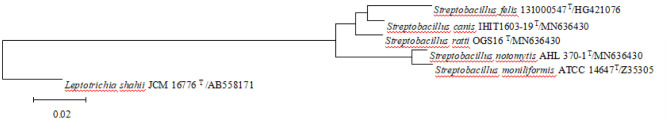
Neighbor-joining phylogenetic tree based on 16S rRNA gene sequences of *Streptobacillus* species. The sequences were compared with *Leptotrichia shahii* JCM 16776^T^ as the out-group. The support of each branch was determined from 1000 bootstrap samples. The GenBank accession number for the sequences used for each species is provided after the organism's name.

To date, methods such as PCR and target gene sequencing have been used to accurately identify *Streptobacillus* at the genus and species levels. The genomic G + C content is 24–26 mol% in the genus *Streptobacillus* [8]. In previous studies, two set primers have been used, including S5: 5′-CATACTCGGAATAAGATGG-3′/AS2: 5′-GCTTAGCTCCTCTTTGTAC-3′ [25],[104]–[106] and SbmF: 5′-GAGAGAGCTTTGCATCCT-3′/SbmR: 5′-GTAACTTCAGGTGCAACT-3′ [9],[10],[107] for accurate identification of *Streptobacillus* spp. with amplicon sizes 269 and 1222 bp, respectively. Eisenberg et al. used *gyrB*, *groEL*, and *recA* genes with 16S rRNA gene to identify four novel species *S*. *ratti*, *S. canis*, *S. notomytis*, and *S. felis* in this genus. The combined analysis of these genes showed a better resolution among the *Streptobacillus* species [7]–[10],[59],[67]. Analysis of the *gyrB* gene sequencing is the best gene target for the phylogenetic resolution from other genes [47],[67],[108],[109]. Several studies have used 16S rRNA gene sequencing for accurate identification of *S. moniliformis* at the genus and species level [110]–[113]. Addidle et al. identified *S. moniliformis* in an epidural abscess sample with 16 rRNA gene sequencing [114]. Andre et al. simultaneously identified *S. moniliformis* using PCR with set primers S5 and AS2 from a child and his pet rat [115]. Adam et al. reported *S. moniliformis* using PCR from a fatal case of rat-bite fever specimen's lung, liver, and epiglottis tissue after death. They also simultaneously identified this organism from the oropharynx tissue of his pet rat [116]. Boot et al. established the 16S rRNA gene using the PCR-RFLP technique, which amplifies a 296 bp fragment of the *S. moniliformis* followed by digestion of segments by BfaI restriction endonuclease and they reported that PCR is more sensitive than culture for identification of *S. moniliformis* in animals. Restriction endonuclease fragment patterns were at 177 and 253 bp [104]. It should be mentioned that the PCR-RFLP technique may be suitable for directly identifying organisms in clinical samples without culturing and isolation [32]. Other studies have performed direct PCR on specimens of the crust of the rat bite sites, blister fluid, synovial fluid, epidural abscess sample, heart valve tissue, necrotic tissues of the pulmonary artery, pustular sample, wound, and cardiac tissue. They identified *S. moniliformis* from their specimens [20],[106],[112]–[115],[117]–[125]. Mackey et al. used PCR and electrospray ionization followed by mass spectrometry (PCR/ESI-MS) to directly identify *S. moniliformis* in serum and synovial fluid [126]. Zhang et al. identified *S. moniliformis* by meta-next generation sequencing (mNGS) and 16S rRNA gene sequencing on the pustular sample [106]. Fukushima et al. identified *S. notomytis* isolated from blood and pustule specimens with 16S rRNA gene sequencing [16]. Kawashima et al. also identified *S. notomytis* with the sequencing of the 16S rRNA gene. Additionally, they simultaneously identified this bacterium in rat feces in their patient's home with next-generation sequencing [127]. Ogawa et al. identified *S. notomytis* by sequencing *groEL*, *gyrB*, and 16S rRNA genes on synovial fluid from Japan. They also identified this bacterium in intraoral specimens in their patient's house rats by nested PCR [11]. Cross-reactivity and similarity have been demonstrated between sequences *Leptotrichia* spp. and *S. moniliformis*; therefore, when PCR is used for accurate identification, it may lead to false positives [128],[129], accordingly, analysis of amplicon sequencing is necessary. Boot et al. amplified a fragment of 296 bp to identify *S. moniliformis* and reported that this fragment has proper sensitivity for identification. However, due to sequence similarity, this fragment can amplify other bacteria, such as *Fusobacterium necrogenes*, *Leptotrichia* spp., and *Sebaldella termitidis* [47],[104],[128]. The similarity between 16S rRNA gene sequences in *S. ratti*, *S. notomytis*, *S. felis*, and *S*. *moniliformis* is from 97.5 to 98.6%. The sequence analysis of the 16S rRNA gene of *Streptobacillus* species indicated that sequence identities between species range from 84.9% to 91.8% [67]. Eisenberg et al. established a multiple-locus VNTR analysis (MLVA) scheme that is species-specific without requiring prior cultivation of the bacteria [130],[131]. Passarett et al. optimized a TaqMan probe-based real-time RT-PCR assay using two target-specific oligonucleotide probes that identified *gyrB* and 16S rRNA target genes to detect *S. moniliformis* in whole blood. They reported that this assay is suitable for accurately identifying this bacterium in clinical laboratories within 3 hr [132]. Another study by Kelly et al. presented a real-time multiplex PCR assay (Target genes: *rpiL* and *grpE*) that can directly identify all species in the genus *Streptobacillus* in clinical specimens including blood, serum, and urine [133]. Also, Fawzy et al. optimized a real-time quantitative (q) PCR (Target genes: 16S rRNA and *gyrB*) for the detection of *S. moniliformis* from clinical specimens of wild rats [134]. Eisenberg et al. reported that the DNA-DNA hybridization (DDH) technique is unsuitable for the differentiation of species in this genus, and the results of this technique are weak [47],[60]. Theodore et al. also reported that polyacrylamide gel electrophoresis (PAGE) is a suitable method for identifying L-forms [135]. Matt et al. identified *S*. *felis* from purple skin lesions with sequencing of 16S rRNA and *gyrB* genes [17]. A phylogenetic tree based on 16S rRNA gene sequences of *Streptobacillus* species is shown in Figure 1.

## 6. Genome features

Due to the genome sequences of five species in the database, phylogenetic analysis was performed based on high-resolution core genome sequences of *Streptobacillus* species. Genome blast distance phylogeny (GBDP) revealed that *S. moniliformis* and *S. ratti* are closely related (Data not shown). Average nucleotide identity (ANI) is a computational analysis that establishes the taxonomic position of the closely related *Streptobacillus* species from other species in this genus with a cut-off point of 95–96% [136]. The pan-genome analysis of the *S. ratti*, *S. notomytis*, *S. felis*, *S. moniliformis*, and *S. canis* comprises 1437, 2130, 1667, 1570, and 1644 predicted genes, respectively (Table 2). The genomic features of these species are shown in Table 2.

**Table 2.** Genomic features of *Streptobacillus* species

Name of organism	Strain	NCBI RefSeq assembly	Genes (total)	Genome size (Mb)	Depositor or source
*Streptobacillus canis*	IHIT1603-19^T^	GCF_009733925.1	1644	1.6	NCBI
*Streptobacillus felis*	131000547^T^	GCF_001559775.1	1667	1.6	NCBI
*Streptobacillus moniliformis*	DSM 12112^T^	GCF_000024565.1	1570	1.7	NCBI
^●^ *Streptobacillus notomytis*	AHL 370-1^T^	GCF_001612575.1	2130	1.7	NCBI
*Streptobacillus ratti*	OGS16^T^	GCF_001891165.1	1437	1.4	NCBI

^●^The genome status of the type strain is suppressed in the GenBank database

## 7. Cellular fatty acid patterns

In 1996, Kämpfer and Kroppenstedt developed a fatty acid methodology [137]. Pins et al. [89], Rowbotham [138], Rygg & Bruun [139], Edwards and Finch [95], Holroyd et al. [80], and Eisenberg et al. [7] also analyzed cellular structural components of *S. moniliformis* by gas-liquid chromatography (GLC) and reported major cellular fatty acids patterns, including C_16:0_ (palmitic acid), C_18:0_ (stearic acid), C_18:1v9c_ (oleic acid), and C_18:2_ (linoleic acid) in 1996, 1983, 1992, 1986, 1988, and 2016, respectively. Torres et al. and Frans et al. identified three peak characteristics, including C_16:0_, C_18:0_, and C_18:1_ of *S. moniliformis* [79],[100]. Another study conducted on *S. moniliformis* using GLC showed that the major fatty acids were C_14:0_, C_16:0_, C_18:2_, C_18:l_, C_18:0_, C_21:0_, C_22:0_, C_24:l_, C_26:l_, and C_20:4_ [139]. Analysis of cellular fatty acid patterns of the *Streptobacillus* spp. are listed in Table 3.

**Table 3.** Comparison of cellular fatty acid composition in the *Streptobacillus* spp.

Fatty acid	Species				
	*Streptobacillus moniliformis* DSM 12112^T^ [7]	*Streptobacillus ratti* DSM 101843^T^ [7]	*Streptobacillus felis* 131000547^T^ [8]	*Streptobacillus notomytis* AHL 370-1^T^ [10],[47]	*Streptobacillus canis* DSM 110501^T^ [9]
C_14 :0_	1.5	1.5	1.5	1.6	0.9
*iso*-C_15:0_	3.9	-	2.1	-	-
C_16:0_	27.8	28.7	28.2	32.5	13.7
C_17:0_	1.5	1.5	1.5	1.5	1.8
C_18:1ω6c_	2.2	5.9	2.0	-	-
C_18:1ω9c_	25.1	23.6	24.1	24.6	12.4
C_18:0_	23.5	26.3	21.6	31.9	25.6
C_20:4ω6,9,12,15c_	1.2	-	1.1	-	1.4

## 8. Serologic identification

Antibody production against *S. moniliformis* has been identified by agglutination and complement fixation tests in guinea pigs, mice, and rats [140]. Enzyme-linked immunosorbent assay (ELISA) and the indirect immunofluorescence assay (IFA) have replaced these assays. In 1993, Boot et al. reported a cross-reaction between *S. moniliformis* and *Acholeplasma laidlawii* in ELISA and IFA assays [140]. The fluorescence in situ hybridization assay (FISH) was used to identify *Fusobacterium* spp. and revealed cross-reaction with *Leptotrichia* spp. and *S. moniliformis* [141]. Immunoblots assay of whole cell antigens of *S. moniliformis* showed different bands in the 32–55 kilodaltons (kD) [33]. Graves and Janda optimized the fluorescent antibody technique with a polyclonal antibody to identify *S. moniliformis* [12]. Generally, serologic tests would help to improve the diagnosis of *S. moniliformis*. Moreover, no seroprevalence study has been conducted on *Streptobacillus* spp. In 2024, Mathé et al. reported an increase in IgM and IgG titers in a 32-year-old woman from Germany suffering from endocarditis caused by *S. moniliformis* [142]. Syphilis-specific serology tests for streptobacillosis were negative [69], but in a few studies, it has been reported that these tests are positive in more than 25% of the patients with this infection [143].

## 9. Virulence factors

Recently, despite genome sequencing of *S. moniliformis*, *S. ratti*, *S. felis*, *S. notomytis*, and *S. canis*, they have not been described as virulence-associated genes. *S. moniliformis* and *S. felis* have an α-hemolytic characteristic, which probably has possible pathogenic properties [35],[47]. Clinical isolates causing severe or fatal infections were non-hemolytic, and these bacteria probably produced other virulence factors such as DNase, which is released growth-independent from the proliferation of bacteria [47]. In the genus *Streptobacillus* cell wall structure, especially *S. moniliformis*, lipopolysaccharide probably plays a major role in pathogenesis [35]. In vitro, *S. moniliformis* can agglutinate blood in animals such as chickens, guinea pig, human, pigs, rats, and turkeys [15],[144]. Microscopic examination showed that *S. moniliformis* can grow within the mice phagocytes; staining with eosin Y also showed that the macrophages ingested *S. moniliformis* were dying significantly faster than macrophages under the same conditions but without streptobacilli [145]. Savage reported that the bacillary forms of *S. moniliformis* are more pathogenic than the coccoidal forms [145], and Freundt revealed that the L-form of *S. moniliformis* has a virulent characterization [91].

## 10. Clinical symptoms

*S. moniliformis* is a zoonotic bacterial pathogen causing Haverhill fever and RBF [10],[15]. The clinical symptoms in RBF caused by *Streptobacillus* occur between 3 days and 4 weeks, but in most patients, symptoms appear within seven days of exposure [20]. The incubation period in RBF for skin lesions or rash ranges from 3 to 21 days [20]. The clinical signs of human infections in RBF are flu-like and non-specific, including soft tissue abscess, epidural abscess, fatigue, fever, diarrhea, malaise, muscle pain, petechial or pustular rash especially on palms and soles, pharyngitis, vomiting [12],[15],[59],[62],[69],[110],[146]–[148], and headache [20],[62],[146]. In many patients, maculopapular, petechial, or purpuric rash can develop into hemorrhagic and pustule vesicular lesions. Also, many patients develop migratory polyarthralgias [20],[146]. Without treatment, *S. moniliformis* and *S. notomytis* infection cause severe septic polyarthritis [67],[149]. Other complications associated with RBF ranging from mild to severe include hepatitis, endocarditis, bacteremia, pericarditis, parotitis, amnionitis, myositis, epiduritis, splenic or renal infarction, pancreatitis, osteomyelitis, nephritis, meningitis, septic arthritis, and brain abscess. These complications occur due to sepsis [10],[15],[76],[131],[150]–[154]. The rat bite wound is often promptly healed [155] without suppurative inflammation and significant regional lymphadenopathy. The most fatal form of complications caused by this disease is endocarditis (Nearly 50%) [20],[75],[156]–[160], and a large number of cases reported had pre-existing various valvular disorders [75], prosthetic heart valves [112], or congenital heart disease [161]. In past studies, this complication's mortality rate was around 53% [12],[15],[162],[163]. Diagnosed confirmed cases of endocarditis due to RBF are as follows: having a history of rodent bite and being positive for vegetations with echocardiography, isolation *S. moniliformis* of blood culture, or positive polymerase chain reaction results [75]. Crofton et al. reported severe recurrent endocarditis due to *S. moniliformis* in a 24-year-old pregnant woman from the USA in 2020 [122]. The clinical presentation of arthritis in RBF varies widely, manifesting in joints of different sizes, such as monoarticular or polyarticular, and exhibiting acute or sub-acute characteristics [1]. Two mechanisms have been proposed to cause arthritis in this disease: one is immunological, and the other is septic arthritis [66]. The knee and hip joints are the most common joints in adult and pediatric cases, respectively [147],[164],[165]. Radiologic findings can be used for diagnosing and managing patients; however, they are not definitive [166]. If clinicians suspect RBF arthralgia to stem from vertebral osteomyelitis or septic arthritis, prompt initiation of antibiotic treatment is imperative [20],[167]. In synovial fluid analysis, white blood cells were high in almost all cases of *S. moniliformis* and *S. notomytis* infections, while neutrophils are the predominant cell types [63],[64],[66],[80],[120],[126],[149],[160],[168]–[170]. To distinguish streptobacillary septic arthritis from reactive arthritis of RBF, using arthrocentesis may help reduce the optimal duration of treatment [1]. Pins et al. reported *S. moniliformis* from a case with an intrauterine device that presumably increased the risk of infection [89]. The clinical features of infections due to *S. notomytis* are similar to infections due to *S. moniliformis* as follows: the presence of arthritis, arthralgia, fever, rash, and sepsis; similarity in antibiotic sensitivity patterns; infection occurs in healthy individuals, and sometimes the absence of puncture marks at the bite site [84]. In various case reports of RBF, the use of non-steroidal anti-inflammatory drugs (NSAIDs) and corticosteroids in treatment protocol worsened the general condition of the patient or led to hospitalization [118],[171]. Due to the corticosteroids suppressing the immune system, their use in the treatment protocol of RBF needs further investigation. In some case reports, the patient had no history of direct contact with rodents or no lesion at the site on admission due to healing, and the origin of the infection was unknown [12],[45],[64],[79],[80],[84],[94],[166],[172]–[177]. As the serologic identification section stated, serological tests would help improve the diagnosis and give an idea of the seroprevalence. *S. ratti* and *S. canis* have not been reported in human infections.

## 11. Haverhill fever

The second type of *S. moniliformis* infection is Haverhill fever which directly or indirectly occurs in humans by eating food or drinking water and milk contaminated with rat urine or oral secretions of rats that are colonized [12],[46],[156],[178]–[180]. Haverhill fever was first recognized in a foodborne outbreak in Haverhill, Massachusetts-USA in 1926 [181] and has been described by three large outbreaks (Haverhill, Massachusetts-USA, Chester-USA, and Chelmsford-England) of this disease. The source of the infection in Haverhill, Massachusetts, and Chester outbreaks may be contaminated raw milk, and in Chelmsford, water was contaminated with rat secretions. In microbiological examination of milk and water samples, *S. moniliformis* was not isolated [78],[181]–[183]. So, this disease is associated with rubellaform to morbilliform (Measles-like) rash and arthritis, and it is also called erythema arthriticum epidemicum [1],[182]. Other names of the disease in literature include streptobacillary RBF, streptobacillosis, and spirillary fever [184]. The onset of the disease in humans is sudden. Clinical signs of Haverhill fever include fever, chills, pharyngitis, headache, sore throat, nausea, and vomiting. Also, it may be accompanied by cough, polyarthralgia, and skin rash (often appears on the hands and feet) [15],[78],[182]–[184]. In patients with septicemia due to Haverhill disease, differential diagnoses from other diseases such as tularemia and brucellosis, are necessary [185]. In the case of Haverhill fever, there is no evidence of person-to-person transmission of infection, and the epidemiological characteristics offered an outbreak of a common source [78]. In America, the incubation period for the milk-borne outbreak of Haverhill fever was 1 to 4 days [78]. There are reports where *S. moniliformis* was isolated from clinical specimens such as blood in Haverhill fever patients [78],[186].

## 12. Differential diagnoses

Fever, rash, and arthritis constitute the classical symptoms of RBF, yet our understanding of seroprevalence remains limited, leaving mild cases and asymptomatic infections largely undetected. Consequently, while the triad fever, rash, and arthritis can prompt diagnosis, other clinical manifestations may not necessarily align. Also, fever, rash, and arthritis may be imitators in drug reactions, noninfectious inflammatory conditions (such as psoriasis, leukocytoclastic vasculitis, bowel-associated dermatosis, and acute generalized exanthematous pustulosis), viral infections (such as hand, foot, and mouth disease, cytomegalovirus, EBV, varicella-zoster virus, Parvovirus B19, and HIV), vasculitis (Henoch-Schönlein purpura, ANCA-associated vasculitis, etc.), rheumatologic (such as spondyloarthropathy, systemic lupus erythematosus, SAPHO syndrome, an drheumatoid arthritis), and bacterial infections (STIs) (such as disseminated gonorrhea, secondary syphilis, Japanese spotted fever, ehrlichiosis, rickettsial disease, brucellosis, and Reiter's syndrome) [20],[80],[85],[105],[117],[146],[147],[152],[164],[166],[177],[187],[188]–[191].

## 13. Review of English literature

The author has reviewed all of the English language articles and abstracts for cases of RBF that happened due to the genus *Streptobacillus* from 1915 to 2023, and a total of 212 cases were reviewed in this study (*S*. *moniliformis*: 206 cases, *S. notomytis*: 5 cases, and *S. felis*: 1 case). The author analysed all cases (Tables S1–S3); the outcomes are discussed below. Analysis of patients' ages indicated the range was from 1 week to 94 years. Also, 56.4% were reported as male among the rat bite patients, and 43.5% were reported as female. Patients' jobs described in these cases are as follows: working in a laboratory (5 cases), student (8 cases), welder (1 case), real estate appraiser (1 case), typist (1 case), insurance rater (1 case), physician (1 case), miner (1 case), farmer (11 cases), an apartment superintendent (1 case), auto mechanic (1 case), warehouse forklift operator (1 case), electrician (1 case), assistant at a veterinary clinic (1 case), pet shop employee (3 cases), retired nurse (1 case), housewife (1 case), homeless (3 cases), owned a bicycle shop (1 case), snake keeper (1 case), bus driver (1 case), working in a mail distribution center (1 case), industrial worker (1 case), and businessman (1 case). Based on the analysis of case reports, the clinical presentation of this condition may manifest with nonspecific symptoms, including fever (78%), rash (51.6%), arthritis (23.4%), or arthralgia (23.9%). Among the 188 cases that were reported reasons for RBF, 144 cases were related to rodents' biting. Biting with rat, pet rat, gerbil, weasel, hamster, and rabbit described were 86/188 (45.7%), 18/188 (9.57%), 1/188 (0.5%), 1/188 (0.5%), 1/188 (0.5%), and 1/188 (0.5%), respectively. Some of these strains have not been adequately investigated concerning bacterial species. After 2010, most of the reports of RBF were related to pet rats (Tables S1–S3), probably due to keeping rats as pets. The range of leukocytosis was 10000–33700 cells/µl, and the dominant cells were polymorphonuclear (Neutrophils) and band cells. According to the analysis of data from tables S1–S3, the percentages of anemia and thrombocytopenia were 20.5% and 4.3%, respectively. The average erythrocyte sedimentation rate (ESR) was 61.11 mm per hour, and cases reported that seven patients had ESR rates below 20 mm per hour. The mortality rate caused by streptobacillosis is rare because many cases exhibited a mild clinical course and reasonable response to antimicrobial therapy. In the literature review carried out by the author, twenty-four (12.2%) of the patients died, and 16 of 24 patients had endocarditis (Tables S1–S3). Complications included endocarditis, pneumonia, bronchitis/otitis, bacteremia, appendicitis, amnionitis, meningitis, septic arthritis, septicemia, synovitis, systemic vasculitis, osteomyelitis, pericarditis, polyarteritis nodosa, focal abscesses, etc. (Tables S1–S3). The mortality rate was high in endocarditis due to infection with the *S. moniliformis* of other complications, with a range of 36.3%. Most cases of endocarditis have been reported in adults that are associated with rash and fever. Endocarditis has not been reported with *S. notomytis* and *S*. *felis*. Treponemal, rheumatoid factor (RF), and antinuclear antibody (ANA) were positive for 6, 2, and 2 cases, respectively. Growth characteristics, isolation, and identification are discussed separately (See the section “isolation-identification” above).

## 14. Antibiogram and treatment

### 14.1. S. moniliformis

Although *S. moniliformis* is a Gram-negative bacterium, it is susceptible to many antibacterial agents that are effective on Gram-positive bacteria [95],[192]. Penicillin is the first-line drug to treat RBF and HF [15],[193]. Also, other antibiotics such as ampicillin, amoxicillin, amoxicillin-clavulanate, second and third-generation cephalosporins, carbapenems, cephalexin, cefuroxime, vancomycin with gentamicin, erythromycin, streptomycin, chloramphenicol, cephalothin, clindamycin and, tetracyclines may be effective for the treatment of this disease [19],[20],[27],[28],[38],[100],[139],[146],[152],[156],[179],[194],[195] or use in patients with a history of a penicillin resistance or allergy existence [20],[66],[103],[112],[122],[196]–[198], However, optimization of durations of standard treatment is necessary. In antimicrobial sensitivity testing, most *Streptobacillus* strains have been reported to be resistant to aminoglycosides, norfloxacin, nalidixic acid, polymyxin B, trimethoprim, and cotrimoxazole [38],[64],[74],[95],[100],[139]. In some reports, treatment failure with erythromycin has been reported [74],[148]. The use of antibiotics orally or in injection depends on the disease's severity and the patient's characteristics. Aminoglycosides have not been recommended for treating arthritis due to streptobacillosis without bacteremia because diffusion of these antibiotics into synovial fluid is poor [147]. In some reported cases, patients with septic arthritis due to streptobacillosis revealed good results in the treatment with clindamycin, flucloxacillin, nafcillin, rifampin, and vancomycin and all these patients recovered from infection [147]. There is no validated clinical breakpoint of antimicrobial susceptibility testing for this genus of bacteria. Eisenberg et al. used the broth micro dilution method for *S*. *ratti* and *S. moniliformis* [7],[8]. In their study, they used antibacterial agents such as amoxicillin/clavulanic acid, ampicillin, ceftiofur, cephalothin, enrofloxacin, erythromycin, florfenicol, gentamicin, penicillin G, spectinomycin, tiamulin, tilmicosin, tetracycline, trimethoprim/sulfamethoxazole, tulathromycin for the survey antimicrobial susceptibility in *S*. *ratti* and MICs (µg/mL) as follows: <2/1, ≥0.25, ≤0.25, <1, ≥1, ≥2, ≥1, <1, ≥0.5, <0.0625, ≥4, <8, <1, ≤0.125, <0.25/4.75, and <2, respectively [7]. Also, they reported resistance to trimethoprim/sulfamethoxazole and sensitivity to azithromycin, ciprofloxacin, clindamycin, chloramphenicol, erythromycin, gentamicin, meropenem, nalidixic acid, streptomycin, telithromycin, and tetracycline in *S. moniliformis* [8]. Kämmerer et al. performed antibiotic susceptibility testing by the E-test method, revealing that their isolate was fully susceptible to cefuroxime and penicillin G with MICs of 0.047 and 0.008 µg/mL, respectively [194]. Addidle et al. reported MICs with the E-test method for ceftriaxone and penicillin 0.006 and 0.012 µg/mL respectively [114]. Adams et al. reported MIC of one strain of *S. moniliformis* by broth microdilution method as follows: ampicillin: < 0.12 µg/mL, ceftriaxone: < 0.06 µg/mL, and gentamicin: > 4 µg/mL [195]. Sakalkale et al. used the disk diffusion method for AST and reported that their isolate was susceptible to ceftazidime, erythromycin, meropenem, piperacillin, and tetracycline but resistant to cotrimoxazole and norfloxacin. Also, they reported that the MIC of penicillin by E-test was 0.008 µg/mL [199]. Balakrishnan et al. used the disc diffusion method for AST, and they reported that their isolate was susceptible to amoxicillin, ceftriaxone, cephalexin, erythromycin, gentamicin, and penicillin G [161]. Results analysis of many studies in the disk diffusion method (diameters of zones) have been interpreted according to DIN 58940 [47]. Also, some authors have interpreted their results based on the cut-off value of the Enterobacteriaceae family with standards for antimicrobial susceptibility testing M100-S24-2014 [200]. According to the literature, resistance to penicillin has not been reported in vitro and in vivo conditions.

### 14.2. S. notomytis

Kawashima et al. performed AST using the micro broth dilution method for *S. notomytis* and the results of MIC was clindamycin < 0.06 µg/mL, cefazolin < 0.5 µg/mL, ampicillin < 0.12 µg/mL, imipenem 0.25 µg/mL, oxacillin < 0.12 µg/mL, levofloxacin < 0.5 µg/mL, and vancomycin < 0.5 µg/mL [127]. Ogawa et al. determined AST by microbroth dilution for one isolate of *S. notomytis* and MIC was as follows cefazolin < 0.5 µg/mL, ceftriaxone 0.25 µg/mL, clarithromycin 8 µg/mL, levofloxacin < 1 µg/mL, minocycline < 0.12 µg/mL, penicillin < 0.06 µg/mL, and vancomycin < 0.25 µg/mL [11]. Another study by Fukushima et al. using the disk diffusion method reported that *S. notomytis* was susceptible to ampicillin, penicillin, cefotaxime, ceftriaxone, imipenem, clindamycin, intermediate to amoxicillin, amoxicillin-clavulanate, gentamicin, erythromycin, doxycycline, minocycline, ciprofloxacin, and resistant to cotrimoxazole [16]. Our literature review for AST showed there are few studies of the AST about *S. notomytis* and this bacterium similar to *S. moniliformis* is susceptible to beta-lactam, macrolide, fluoroquinolone/quinolone, aminoglycoside, and tetracycline antibiotics (Table S2).

## 15. Geographic distribution/epidemiology

In our literature review, RBF due to *Streptobacillus* spp. has been reported in the USA (91 cases), Australia (6 cases), Irland (1 case), Norway (2 cases), Scotland (1 case), UK (20 cases), Canada (9 cases), Israel (3 cases), Netherlands (1 case), Spain (6 cases), France (10 cases), Greece (2 cases), Switzerland (2 cases), Belgium (2 cases), Singapore (1 case), Thailand (2 cases), India (2 cases), Taiwan (2 cases), Hong Kong (1 case), New Zealand (2 cases), Germany (4 cases), Japan (12 cases), Denmark (3 cases), Kuwait (1 case), Qatar (2 cases), China (2 cases), Portugal (1 case), Hawaii (1 case), and Poland (1 case) (Tables S1, S2, S3). There are few reports from Africa with *S. moniliformis* in the literature. In 1916, Blake first reported an infection with *Streptobacillus* in the United States [70]. In the USA, Referrals to the emergency department for RBF is 0.33 per one million individuals, and 60% of cases diagnosed are hospitalized for further management [201]. It is estimated that of 40,000 rat bites annually [15],[202], 2% lead to human infection [203]. Annually, 20,000 rat bites are registered in the USA [204]. In the USA, more than 30 percent of the instances of RBF have been reported in children with age less than 15 years old [18],[27], and the primary cause of this disease is *S. moniliformis* [205]. Because of the popularity of rats as pets, infections with this bacterium are increasing in individuals, especially children [206]. In literature, the mortality rate is different in patients with untreated RBF and has been reported as 10% [78], 13% [12],[20],[38], and more than 25% [85],[156].

## 16. Prevention of RBF

It is necessary to make people aware of the possible dangers of water and food contaminated with excreta or oral secretions of rodents [207]. Laboratory animal workers and owners of rodents should wear protective instruments, such as gloves and protective clothing, and after contact with rodents or cage cleaning, avoid touching the face, specifically the mouth, and wash their hands and face with water and soap [208],[209]. As long as humans and rats live together, it is probably impossible to completely prevent RBF. In urban regions, rodent eradication programs are beneficial, but these programs could be more effective and economical for rural areas. Effective prophylactics of RBF in the absence of increased antibiotic resistance with penicillin or other antibiotics is unknown and further studies are required on this topic.

## 17. Conclusions

The most common clinical signs of human infections in RBF include fever, maculopapular, and petechial or pustular rash. However, *S. moniliformis* and *S. notomytis* are amazingly susceptible to various antibiotics, especially penicillin. Many patients experience therapy delays due to nonspecific clinical manifestations, a list of broad differential diagnoses, and difficulties in microbiological diagnosis. Asking questions about the rodent bite history and completing a skin examination is necessary when evaluating a fever of unknown origin because patients perhaps forget it or consider it unimportant (The transmission route is mainly unknown). Also, seroprevalence and zoonotic reservoirs remain largely unknown, and more studies need to be done on these topics. Whole-genome sequencing (WGS) of *Streptobacillus* spp. can provide new information about their genomic characteristics. The author recommends optimizing regional reference laboratories in each region to diagnose patients' suspicious cases with RBF. Finally, a delay in the diagnosis of infection affects the proper and timely treatment of the patient and may even endanger the patient's health.


