# Supplementary material for: Rat-Bite Fever due to the genus Streptobacillus
Source: AIMS Microbiol. 2024 Nov 5;10(4):917–43. doi: 10.3934/microbiol.2024040 (PMC11609424; doi:10.3934/microbiol.2024040)
Supplement: Supplementary file 1 [file microbiol-10-04-040-s001.pdf]

AIMS Microbiology

AIMS Microbiology, 10 (4): 917–943.
DOI: 10.3934/microbiol.2024040
Received: 08 February 2024
Revised: 10 July 2024
Accepted: 14 October 2024
Published: 05 November 2024

http://www.aimspress.com/journal/microbiology

Review

Rat-Bite Fever due to the genus *Streptobacillus*

Mehdi Fatahi-Bafghi[1,2,*]

[1] Infectious Diseases Research Center, Shahid Sadoughi Hospital, Shahid Sadoughi University of Medical Sciences, Yazd, Iran
[2] Department of Microbiology, Faculty of Medicine, Shahid Sadoughi University of Medical Sciences, Yazd, Iran

* **Correspondence:** Email: mehdifatahi371@gmail.com; mehdifatahi@ssu.ac.ir.

Supplementary

Table S1. Summary of published case reports on *S. moniliformis*.

Patient age/Gender	Year of publication	country	Potential contact with rats or job	Bite site/ Rodent type	Clinical signs	RBC, HB, HCT%, WBC count per uL/Crp per mg/L /ESR mm/h	Complications/Isolated from	Treponemal test/RF/ANA	Identification method	Affected joints	ALT(IU/L)/AST (U/L) /UREA/CREATININ/GGT (U/L) /LIPAS/AMILASE	Antibiogram method/ Antibiotic treatment	Outcome	Ref
67/F	1915	USA	-	Finger/ Rat	Fever, maculopapular rash, joint pain, apical systolic murmur, chill	-/-/-/22100/-/-	Endocarditis /Blood culture	N/-/-	Phenotypic characterization, inoculation of animals	-	-/-/-/-/-/-/-	-/-	Died	[1-3]
46/F	1916	USA	-	Toe/Rat	Rash, dysphagia, fever, vomiting, headache, swelling, erythema, mitral murmur, anemia, lymphadenopathy	-/-/-/24000/-/-	-/Blood culture	N/-/-	-	-	-/-/-/-/-/-/-	-/Potassium arsenite	Cure	[1,4]
5/M	1916	USA	-	-/Rat	Rash, muscle aching	-/-/-/-/-/-	-/-	-/-/-	-	-	-/-/-/-/-/-/-	-/-	-	[1,5]
14/M	1917	USA	-	-/Rat	Rash	-/-/-/-/-/-	-/-	-/-/-	-	-	-/-/-/-/-/-/-	-/-	-	[1,5]
10/M	1918	USA	-	Thumb/ Weasel	Rash, swelling, lymphadenopathy, fever, malaise, vomiting	-/-/-/19000/-/-	-/Blood culture	+/-/-	Phenotypic characterization, serologic method	-	-/-/-/-/-/-/-	-/	Cure	[1,6]
13/M	1918	USA	-	Face /Rat	Rash, lymphadenopathy, anemia	4200000/-/-/32000/-/-	-/Blood culture	N/-/-	Phenotypic characterization, serologic method	-	-/-/-/-/-/-/-	-/-	Died	[1,7]
10/M	1926	USA	-	-/Rat	Rash, arthritis	-/-/-/-/-/-	-/-	-/-/-	-	-	-/-/-/-/-/-/-	-/-	-	[1]

Continued on next page

Patient age/Gender	Year of publication	country	Potential contact with rats or job	Bite site/ Rodent type	Clinical signs	RBC, HB, HCT%, WBC count per uL/Crp per mg/L /ESR mm/h	Complications/Isolated from	Treponemal test/RF/ANA	Identification method	Affected joints	ALT(IU/L)/AST (U/L) /UREA/CREATININ/GGT (U/L) /LIPAS/AMILASE	Antibiogram method/ Antibiotic treatment	Outcome	Ref
-/M	1934	USA	-	-/Rat	Rash, arthritis	-/-/-/-/-/-	-/-	-/-/-	-	-	-/-/-/-/-/-/-	-/-	-	[1,8]
18/M	1934		-	-/-	Incomplete description	-/-/-/-/-/-	Endocarditis /Blood culture	-/-/-	-	-	-/-/-/-/-/-/-	None	Died	[2]
18/M[*1]	1935		-	-/-	Incomplete data; anemia	-/-/-/-/-/-	Endocarditis /Blood culture	-/-/-	-	-	-/-/-/-/-/-/-	-/None	Died	[9,10]
40/M	1939	USA	-	-/Rat	Rash, arthritis	-/-/-/-/-/-	-/-	-/-/-	-	-	-/-/-/-/-/-/-	-/-	-	[1]
3 monthold/F	-	USA	-	-/Rat	Rash, arthritis	-/-/-/-/-/-	-/-	-/-/-	-	-	-/-/-/-/-/-/-	-/-	-	[1]
-/M	1939	USA	-	-/Rat	Rash, arthritis	-/-/-/-/-/-	-/-	-/-/-	-	-	-/-/-/-/-/-/-	-/-	-	[1,11]
-/M	1939	USA	-	-/Rat	Rash, arthritis	-/-/-/-/-/-	-/-	-/-/-	-	-	-/-/-/-/-/-/-	-/-	-	[1,11]
21/M	1940	USA	Working in a medical laboratory	Thumb /Rat	Maculopapular and petechial rash, arthritis, malaise, chill, fever, lymphadenopathy, anemia	-/8/-/32000/-/-	-/Blood culture	N/-/-	-	Knee, elbow, shoulder, hip	-/-/-/-/-/-/-	-/Arsenic	Cure	[1,12]
14/F	1940	-	-	-/Rat	Apical systolic murmur, petechial and maculopapular rash, anemia	-/-/-/-/-/-	Endocarditis / Heart valve	-/-/-	-	-	-/-/-/-/-/-/-	None	Died	[2,13]
21/M	1941	USA	-	-/Rat	Rash	-/-/-/-/-/-	-/-	-/-/-	-	-	-/-/-/-/-/-/-	-/-	-	[1]
14/F	1941	Australia	-	Face/ Rat	Fever, macular and petechial rash, abdominal pain, joint and muscle pains, apical systolic murmur, anemia	-/-/-/18600/-/-	Endocarditis /Blood culture	-/-/-	Phenotypic characterization, inoculation of animals	-	-/-/-/-/-/-/-	-/None	Died	[10, 14]
49/M	1942	USA	-	-/Rat	Rash, arthritis	-/-/-/-/-/-	-/-	-/-/-	-	-	-/-/-/-/-/-/-	-/-	-	[1,15]
49/M	1942	USA	-	-/Rat	Rash	-/-/-/-/-/-	-/-	-/-/-	-	-	-/-/-/-/-/-/-	-/-	-	[1,15]

Continued on next page

Patient age/Gender	Year of publication	country	Potential contact with rats or job	Bite site/ Rodent type	Clinical signs	RBC, HB, HCT%, WBC count per uL/Crp per mg/L /ESR mm/h	Complications/Isolated from	Treponemal test/RF/ANA	Identification method	Affected joints	ALT(IU/L)/AST (U/L) /UREA/CREATININ/GGT (U/L) /LIPAS/AMILASE	Antibiogram method/ Antibiotic treatment	Outcome	Ref
21/M	1942	USA	-	-/Rat	Arthritis	-/-/-/-/-/-	-/-	-/-/-	-	-	-/-/-/-/-/-/-	-/-	-	[1,15]
6 monthold/M	1944	USA	-	Finger/ Rat	Swelling, fever, petechial and macular rash, sore throat	3660000/ 64 per cent/-/12200/-/-	-/Blood culture	N/-/-	Phenotypic characterization	Finger	-/-/-/-/-/-/-	-/Sulfadiazine	Cure	[16]
15/M	1944	Ireland	Student	Finger /Rat	Fever, vomiting, headache, loss of appetite, malaise, rash	4120000/-/-/3200/-/30	-/Blood culture	+/-/-	Phenotypic characterization, inoculation of animals	-	-/-/-/-/-/-/-	-/Penicillin	Cure	[17]
73/F	1944	USA	-	Thumb/ Rat	Malaise, fever, purplish-red macular rash, dyspneic, swelling	3660000/12.5/-/21350/-/-	Bronchopneumonia. /Blood culture	-/-/-	-	Thumb	-/-/-/-/-/-/-	-/Sulfadiazine, followed by acetylsalicylic acid	Died	[18]
43/M	1944			Rat exposure	Fever, rash, murmur	-/-/-/-/-/-	Endocarditis /-	-/-/-	-	-	-/-/-/-/-/-/-	-/Penicillin G	Died	
18 months old/F	1945	USA	-	Toe/Rat	Fever, chill, macular pink rash, hepatomegaly, pain in the shoulder	-/-/-/14650/-/-	-/Blood culture	N/-/-	Phenotypic characterization	-	-/-/-/-/-/-/-	-/Penicillin G	Cure	[19]
43/M*1	1945	USA	-	Contact with rats 2 year before present disease	Mitral and aortic double murmurs, fever, night sweats, fatigue, migratory arthralgia, macular rash	-/-/-/-/-/-	Endocarditis /Blood culture	-/-/-	-	-	-/-/-/-/-/-/-	-/Penicillin G	Died	[2,20]

Continued on next page

Patient age/Gender	Year of publication	country	Potential contact with rats or job	Bite site/ Rodent type	Clinical signs	RBC, HB, HCT%, WBC count per uL/Crp per mg/L /ESR mm/h	Complications/Isolated from	Treponemal test/RF/ANA	Identification method	Affected joints	ALT(IU/L)/AST (U/L) /UREA/CREATININ/GGT (U/L) /LIPAS/AMILASE	Antibiogram method/ Antibiotic treatment	Outcome	Ref
7 week old/-	1945	USA	-	Finger and lip/ Rat	Fever, soft green stools, loss of appetite, adenopathy, swelling, arthritis, anemia, sore throat	-/7.6/-/8650/-/-	-/Blood culture	N/-/-	Phenotypic characterization, inoculation of animals	Finger, wrist,	-/-/-/-/-/-	-/Penicillin G	Cure	[21]
5 week old/M*2	1945	USA	-	Finger and lip/ Rat	Fever, swelling of the penis and scrotum	-/-/-/17400/-/-	Bronchitis and otitis/ Blood culture	+/-/-	Phenotypic characterization, inoculation of animals	-	-/-/-/-/-/-	-/Penicillin G	Cure	[21]
6 month old/M*3	1945	USA	-	Finger/ Rat	Fever, macular rash, anemia, swelling	-/ 7.8/-/25000/-/-	-/Blood culture	N/-/-	Phenotypic characterization, inoculation of animals	-	-/-/-/-/-/-	-/Penicillin G	Cure	[21]
2 month old/M	1945	USA	-	Hand and foot/Rat	Fever, maculopapular rash, lymphadenopathy, arthritis	-/-/-/17500/-/-	-/Blood cultures	N/-/-	-	-	-/-/-/-/-/-	Micro broth dilution/ Penicillin G	Cure	[1]
10 montholt d/M	1945	USA	-	Ankle/Rat	Fever, lymphangitis, maculopapular rash, arthritis	-/-/-/13400/-/-	-/Blood cultures	N/-/-	-	-	-/-/-/-/-/-	Micro broth dilution/ Penicillin G	Cure	[1]
4.5/F	1945	USA	-	Hand/Rat	Maculopapular rash, swelling joints, fever, arthritis	-/-/-/9200/-/-	-/Blood culture	N/-/-	-	-	-/-/-/-/-/-	Micro broth dilution/ Penicillin G	Cure	[1]

Continued on next page

Patient age/Gender	Year of publication	country	Potential contact with rats or job	Bite site/ Rodent type	Clinical signs	RBC, HB, HCT%, WBC count per uL/Crp per mg/L /ESR mm/h	Complications/Isolated from	Treponemal test/RF/ANA	Identification method	Affected joints	ALT(IU/L)/AST (U/L) /UREA/CREATININ/GGT (U/L) /LIPAS/AMILASE	Antibiogram method/ Antibiotic treatment	Outcome	Ref
22/M[*1]	1945	-	-	-/-	Mitral systolic, aortic double murmurs, petechial rash	-/-/-/-/-/-	Endocarditis /Blood culture	-/-/-	-	-	-/-/-/-/-/-/-	-/Penicillin G	Died	[2]
48/F	1946	USA	-	Finger /Rat	Fever, swelling, cough, anorexia, papular and petechia rash, night sweat, anemia	3480000/10.7/-/9700/-/-	-/Blood culture	+/-/-	Phenotypic characterization, serologic method	Hip, wrist, elbow, knee, ankle, shoulder	-/-/-/-/-/-/-	-/Penicillin G	Cure	[22]
21/M	1947	USA	Worked in a nutrition laboratory as care of white laboratory rat	Finger /White rat	Fever, headache, chill, swelling, sore throat, backache, arthralgia, rash, systolic murmur	-/-/-/9700/-/2	Bacteremia/ Blood culture	N/-/-	Phenotypic characterization, serologic method, inoculation of animals	Knees, elbows	-/-/-/-/-/-/-	-/Penicillin G	Cure	[23]
38/M	1947	-	Welder	consuming of food or water	Fever, vomiting, nausea, diarrhea, swelling, anemia, weakness, chill, night sweat, cough, weigh lose, valvular cardiac murmurs	4040000/66/-/10000/-/8	Haverhill fever/Blood culture	N/-/-	Phenotypic characterization, serologic method	Knee, ankle	-/-/-/-/-/-/-	Disk diffusion and micro broth dilution/ Streptomycin	Cure	[24]
17/F	1947		-	-/-	-	-/-/-/-/-/-	Endocarditis /-	-/-/-	-	-	-/-/-/-/-/-/-	-/Penicillin G	Died	[13,25]

Continued on next page

Patient age/Gender	Year of publication	country	Potential contact with rats or job	Bite site/ Rodent type	Clinical signs	RBC, HB, HCT%, WBC count per uL/Crp per mg/L /ESR mm/h	Complications/Isolated from	Treponemal test/RF/ANA	Identification method	Affected joints	ALT(IU/L)/AST (U/L) /UREA/CREATININ/GGT (U/L) /LIPAS/AMILASE	Antibiogram method/ Antibiotic treatment	Outcome	Ref
25/F	1948	Norway	working in a laboratory and bitten by a laboratory rat	Finger/ Rat	Chill, vomiting, headache, fever, arthralgia, weakness	4800000/98%/-/10400/-/6	-/Blood culture	-/-/-	Serologic method, inoculation of animals	Axillary	-/-/-/-/-/-/-	-/Sulfathiazole and penicillin	Cure	[26]
-/M	1948	Scotland	-	Thumb/ Rat	Fever, severe pains in the limbs and back, maculo-papular rash	-/-/-/10400/-/-	-/Blood culture	-/-/-	Phenotypic characterization	Elbow	-/-/-/-/-/-/-	Micro broth dilution/ Penicillin G	Cure	[27]
40/M	1949	-	-	Rat exposure; could not recall a rat bite	Anemia, murmur, splenomegaly, Osler's nodes	-/-/-/-/-/-	Endocarditis/-	-/-/-	-	-	-/-/-/-/-/-/-	-/Penicillin G	Cure	[28]
27/M	1949	-	-	Handled a dead rat	Anemia, fever, murmur, splenomegaly, Oslers nodes	-/-/-/-/-/-	Endocarditis/-	-/-/-	-	-	-/-/-/-/-/-/-	-/Penicillin G	Cure	[28]
9/M	1950	USA	-	Finger/ Rat	Cough, macular and petechial rash, swelling, vomiting, nausea, abdominal pain, lymphadenopathy, anemia, weight loss	3910000/11/-/11000/-/76	-/-	N/-/-	-/Serologic method	Limp	-/-/220/-/-/-/-	-/Penicillin G	Died	[29]

Continued on next page

Patient age/Gender	Year of publication	country	Potential contact with rats or job	Bite site/ Rodent type	Clinical signs	RBC, HB, HCT%, WBC count per uL/Crp per mg/L /ESR mm/h	Complications/Isolated from	Treponemal test/RF/ANA	Identification method	Affected joints	ALT(IU/L)/AST (U/L) /UREA/CREATININ/GGT (U/L) /LIPAS/AMILASE	Antibiogram method/ Antibiotic treatment	Outcome	Ref
40/M*1	1950	USA	Real estate appraiser	Thumb and toes/ Rat	Fever, weakness, chill, lymphadenopathy, Present mitral presystolic and systolic and aortic systolic and diastolic murmurs, present duroziez's sign, anemia	3600000/11/-/6500/-/13	Endocarditis /Blood culture	N/-/-	Phenotypic characterization and serologic method	NO	-/-/19.8/-/-/-/-	Micro broth dilution/ Penicillin G	Cure	[2]
27/F*1	1951	UK	Typist	Handled a dead rat	Fever, Osier's nodes, arthralgia, splenomegaly, anemia, murmurs of mitral stenosis and aortic insufficiency, sweat, fatigue, tachycardia	4600000/7.4/-/5600/-/30	Endocarditis /Blood culture	-/-/-	Phenotypic characterization	Knees, metacarpophalangeal, shoulder	-/-/-/-/-/-/-	-/Penicillin G	Cure	[30]
33/M	1951	USA	Insurance rater	Could not recall a rat bite or contact with rodents	Fever, liver tenderness	-/-/-/-/-/-	Appendicitis Subphrenic abscess followed Acute appendicitis /Pus	-/-/-	Phenotypic characterization	-	-/-/-/-/-/-/-	-/Streptomycin and penicillin, followed by aureomycin, streptomycin, and penicillin	Cure	[31]
54/M	1952	-	-	-/Rat	Murmur, fever, rash	-/-/-/-/-/-	Endocarditis /-	-/-/-	-	-	-/-/-/-/-/-/-	-/Penicillin G	Cure	[13]

Continued on next page

Patient age/Gender	Year of publication	country	Potential contact with rats or job	Bite site/ Rodent type	Clinical signs	RBC, HB, HCT%, WBC count per uL/Crp per mg/L /ESR mm/h	Complications/Isolated from	Treponemal test/RF/ANA	Identification method	Affected joints	ALT(IU/L)/AST (U/L) /UREA/CREATININ/GGT (U/L) /LIPAS/AMILASE	Antibiogram method/ Antibiotic treatment	Outcome	Ref
54/M	1953	USA	Physician	Thumb/ Rat	Fever, chill, headache, macular rash, shoulder pain, apical systolic murmur, nausea	4190000/13.5/-/22000/-/-	Endocarditis /Blood culture	-/-/-	Phenotypic characterization, serologic method	-	-/-/-/-/-/-	Micro broth dilution/ Penicillin G	Cure	[32]
3/M	1959	USA	-	Could not recall a rat bite or contact with rodents	Swelling, petechial rash, anemia, lymphadenopathy	-/11.1/-/12000/+/47	-/Blood culture was negative	-/-/-	Serologic method	Knee	-/-/-/-/-/-	-/The patient was dismissed untreated	Cure	[33]
Seven and a half years old/M	1959	USA	-	Could not recall a rat bite or contact with rodents	Fever, swelling	-/12/38/3000/NO/12	-/Blood culture was negative	-/-/-	Serologic method	Knees	-/-/-/-/-/-	-/The patient was dismissed untreated	Cure	[33]
28/F	1964	Canada	Working in a laboratory	Finger/ Rat	Headache, nausea, vomiting, muscle pains, swelling, fever	-/13.6/44/12600/-/-	-/Blood culture	-/-/-	Phenotypic characterization	Ankle	-/-/29/-/-/-/-	Disk diffusion /Tetracycline, followed by penicillin G	Cure	[34]
67/M	1966	UK	Miner	Hand/ Rat	Fever, polyarthritis, sweat, dysuria, low backache, anemia	-/11.3/-/10000/-/129	-/Blood culture	-/-/-	-	Knee	-/-/-/-/-/-	-/Streptomycin, followed by penicillin	Cure	[35]

Continued on next page

Patient age/Gender	Year of publication	country	Potential contact with rats or job	Bite site/ Rodent type	Clinical signs	RBC, HB, HCT%, WBC count per uL/Crp per mg/L /ESR mm/h	Complications/Isolated from	Treponemal test/RF/ANA	Identification method	Affected joints	ALT(IU/L)/AST (U/L) /UREA/CREATININ/GGT (U/L) /LIPAS/AMILASE	Antibiogram method/ Antibiotic treatment	Outcome	Ref
67/M	1966	UK	Farmer	Hand/ Rat	Fever, anorexia, headache, sweat, aching muscular, erythematous rash, polyarthritis	-/15.3/-/13000/-/70	-/Blood culture	-/-/-	-	Shoulder, knees	NO/NO/48/-/-/-/-	-/Streptomycin and benzylpenicillin,	Cure	[35]
43/M	1967	USA	-	Finger/ Rat	Fever, chill, low back pain, erythema, swelling, murmurs of mitral stenosis, arthritis	-/13.8/40/16500/-/-	Pericardial effusion/ Pericardial fluid [Endocarditis]/ Blood culture	-/-/-	Phenotypic characterization, serologic method	Elbow, shoulder	-/-/-/-/-/-/-	-/Penicillin G and chloramphenicol	Died	[36, 37]
70/M*4	1967	USA	-	-/-	Nausea, weight loss, fever, cough, present systolic thrill and a loud systolic murmur at the aortic area, anemia, night sweat, splenomegaly	-/10/-/12300/-/-	Endocarditis /Blood culture	-/-/-	-	-	-/-/-/-/-/-/-	-/Penicillin G	Cure	[10]
60/M*1	1967	USA	An apartment super intended	Worked in a rat-infested area; Could not recall a rat bite	Chill, fatigue, migratory symmetrical arthralgia, night sweats, weight loss, fever, present systolic thrill and a loud systolic murmur at the aortic area	-/13.5/-/14200/-/-	Endocarditis /Blood culture	-/-/-	-	-	-/-/-/-/-/-/-	-/Penicillin G	Cure	[10, 37]

Continued on next page

Patient age/Gender	Year of publication	country	Potential contact with rats or job	Bite site/ Rodent type	Clinical signs	RBC, HB, HCT%, WBC count per uL/Crp per mg/L /ESR mm/h	Complications/Isolated from	Treponemal test/RF/ANA	Identification method	Affected joints	ALT(IU/L)/AST (U/L) /UREA/CREATININ/GGT (U/L) /LIPAS/AMILASE	Antibiogram method/ Antibiotic treatment	Outcome	Ref
44/M	1973	USA	Auto mechanic	Handling infected dead rats	Fever, chill, nausea, vomiting	-/-/-/11200/-/-	Haverhill fever/Blood culture	-/-/-	Phenotypic characterization, serologic method	Shoulder	-/-/NO/-/-/-/-	Broth dilution/ Cephalothin and tetracycline	Cure	[38]
55/F	1976	USA	-	-/-	Anorexia, weakness, myalgia, systolic ejection murmur, hepatomegaly	-/-/-/17900/-/-	Endocarditis /Blood culture	-/-/-	-	-	-/-/-/-/-/-/-	-/ Cephalothin and gentamicin,	Died	[39]
11/M	1979	USA	Rural environment	-/-	Subglottic mass, bilateral parotid swelling, fever, erythematous maculopapular rash, arthritis	-/-/-/-/-/-	-/-	-/-/-	-	Ankle	-/-/-/-/-/-/-	-/Penicillin G	Cure	[40, 41]
23/F	1980	USA	Unknown	-/-	Afebrile and without the did any obvious signs of infection,	-/-/-/-/-/-	Amnionitis /Amniotic fluid	-/-/-	Phenotypic characterization	-	-/-/-/-/-/-/-	-/-	-	[42]
41/M	1981	-	-	-/Rat	Heart failure, fever, murmur	-/-/-/-/-/-	Endocarditis /-	-/-/-	-	-	-/-/-/-/-/-/-	-/Penicillin and gentamicin	Died	[13]
17/F	1983	UK	Student	Consuming raw milk	Fever, malaise, nausea, erythematous macules rash, headache, photophobia	-/-/-/NO/-/-	Haverhill fever/ Blood culture	-/-/-	Phenotypic characterization, serologic method	hip, knee, ankle	-/-/-/-/-/-/-	Agar dilution/ Penicillin G	Cure	[43]

Continued on next page

Patient age/Gender	Year of publication	country	Potential contact with rats or job	Bite site/ Rodent type	Clinical signs	RBC, HB, HCT%, WBC count per uL/Crp per mg/L /ESR mm/h	Complications/Isolated from	Treponemal test/RF/ANA	Identification method	Affected joints	ALT(IU/L)/AST (U/L) /UREA/CREATININ/GGT (U/L) /LIPAS/AMILASE	Antibiogram method/ Antibiotic treatment	Outcome	Ref
16/F	1983	UK	Student	Consuming raw milk	Fever, malaise, shivering, nausea, vomiting, headache, arthralgia, maculopapular rash, sore throat, earache	-/-/-/17300/-/-	Haverhill fever/ Blood culture	-/-/-	Phenotypic characterization, gas chromatography	-	-/-/-/-/-/-/-	Agar dilution/Co-trimoxazole, followed by tetracycline	Cure	[43]
13/F	1983	UK	Student	Consuming raw milk	Fever, malaise, nausea, headache, myalgia, arthritis, sore throat, laryngitis, maculopapular rash, swelling, earache	-/-/-NO/-/-	Haverhill fever/ Blood culture	-/-/-	-	Knee, shoulder, temporomandibular, wrist, elbow, finger	-/-/-/-/-/-/-	Agar dilution/Tetracycline	Cure	[43]
22/F	1983	UK	Student	Consuming raw milk	Headache, myalgia, fever, rigors, vomiting, erythematous rash, arthralgia, sore throat, lymphadenopathy	-/-/-/ NO/-/-	Haverhill fever/ Blood culture	-/-/-	-	Shoulder, knee, hip	-/-/-/-/-/-/-	Agar dilution/ Penicillin G	Cure	[43]
15/F*1	1983	Israel	-	-/-	Fever, chill, apical systolic and an aortic diastolic murmur, petechial rash, hepatomegaly, splenomegaly, anemia	-/9.6/-/20000/-/68	Endocarditis /Blood culture	-/-/-	-	-	-/-/-/-/-/-/-	-/Penicillin G	Cure	[44]

Continued on next page

Patient age/Gender	Year of publication	country	Potential contact with rats or job	Bite site/ Rodent type	Clinical signs	RBC, HB, HCT%, WBC count per uL/Crp per mg/L /ESR mm/h	Complications/Isolated from	Treponemal test/RF/ANA	Identification method	Affected joints	ALT(IU/L)/AST (U/L) /UREA/CREATININ/GGT (U/L) /LIPAS/AMILASE	Antibiogram method/ Antibiotic treatment	Outcome	Ref
28/F	1984	Netherlands	-	Could not recall a rat bite or contact with rodents; consuming unpasteurized milk	Fever, headache, muscle tenderness, meningeal irritation and hemiparesis	-/-/-/11000/-/90	Meningitis/ Material obtained by puncture [parasagittal burr]	-/-/-	Phenotypic characterization	-	-/-/-/-/-/-/-	-/Penicillin G	Cure	[45]
Three-month old/M	1985	USA	-	Hand/ Rat	Fever, lymphadenopathy, hepatic, interstitial pneu monia, splenic congestion, arthritis, anemia, lethargy	-/11.3/34.7/9800/-/-	Endocarditis /Blood culture	-/-/-	-	Several joints	-/-/-/-/-/-/-	-/-	Died	[46]
63/F	1985	-	-	-/Rat	Murmur, fever, arthritis	-/-/-/-/-/-	Endocarditis /-	-/-/-	-	-	-/-/-/-/-/-/-	-/Penicillin and amikacin	Died	[13]
77/M	1985	USA	Farmer	-/Rat	Fever, arthralgia, pustules formation, anorexia	-/-/-/-/-/-	Septic arthritis/Blood culture	-/-/-	-	Proximal interphalangeal, metacarpophalangeal, wrists, ankles	-/-/-/-/-/-/-	-/Cloxacillin and ampicillin, followed by penicillin G	Cure	[47-49]

Continued on next page

Patient age/Gender	Year of publication	country	Potential contact with rats or job	Bite site/ Rodent type	Clinical signs	RBC, HB, HCT%, WBC count per uL/Crp per mg/L /ESR mm/h	Complications/Isolated from	Treponemal test/RF/ANA	Identification method	Affected joints	ALT(IU/L)/AST (U/L) /UREA/CREATININ/GGT (U/L) /LIPAS/AMILASE	Antibiogram method/ Antibiotic treatment	Outcome	Ref
8/F	1986	USA	-	Could not recall a rat bite and or contact with rodents; not consuming contaminated food	Fever, arthralgia, malaise, lethargy, dyspnea, weight loss, palpable thrill at the aortic area, aortic stenosis, anemia, splenomegaly, murmur	-/-/27/12500/-/-	Endocarditis / Blood culture	-/-/-	-	-	-/-/-/-/-/-/-	-/Penicillin G	Cure	[50]
79/M*5	1987	UK	Farmer	Hand/ Rat	Fever, chill, abdominal pain, swelling, anemia, arthralgia, confusion	-/11.5/-/13700/-/-	Septic arthritis/Synovial fluid	-/-/-	Phenotypic characterization	Metacarpophalangeal, knee, wrists	-/-/-/-/-/-/-	-/Penicillin G	Cure	[51]
48/M	1987	USA	Warehouse fork lift operator	Could not recall a rat bite or contact with rodents	Malaise, fever, chill, hoarseness, sore throat, headache, night sweat, cough, arthritis, swelling, arthralgia, myalgia	-/12.9/-/14200/-/130	Septic arthritis/ Joint fluid	N/N/-	Phenotypic characterization	Wrist, shoulder	-/-/-/-/-/-/-	-/Nafcillin and gentamicin	Cure	[49, 52]

Continued on next page

Patient age/Gender	Year of publication	country	Potential contact with rats or job	Bite site/ Rodent type	Clinical signs	RBC, HB, HCT%, WBC count per uL/Crp per mg/L /ESR mm/h	Complications/Isolated from	Treponemal test/RF/ANA	Identification method	Affected joints	ALT(IU/L)/AST (U/L) /UREA/CREATININ/GGT (U/L) /LIPAS/AMILASE	Antibiogram method/ Antibiotic treatment	Outcome	Ref
59/M*12	1988	USA	-	Could not recall a rat bite or contact with rodents	Swelling, fever, arthritis	-/-/-/25600/-/-	Polyarthritis / Blood culture and joint fluid	-/-/+	Phenotypic characterization, gas chromatography	proximal interphalangeal, metacarpophalangeal, wrist, knee, elbows, shoulders	-/-/-/-/-/-/-	Disk diffusion/Ticarcillin and gentamicin, followed by penicillin G	Cure	[48, 53]
39/M	1988	UK	Electrician	Thumb/ Pet gerbil	Sore throat, maculopapular rash, fever	-/NO/-/NO/-/40	Leucocytoclastic vasculitis /Blood culture	+/-/-	Phenotypic characterization	Ankle, elbow, knee	NO/NO/-/-/NO/NO/NO	-/Penicillin G	Cure	[54]
Two-monthold/M	1989	USA	Living in a rural area	Finger/ Rat	Fever, diarrhea, vomiting, swelling, hepatomegaly, splenomegaly, lymphadenopathy	NO/NO/NO/NO/-/-	Endocarditis and meningitis/ CSF	-/-/-	Phenotypic characterization	Wrists, fingers	-/-/-/-/-/-/-	-/-	Died	[13, 55]
54/F	1990	Spain	-	-/Rat scratch	Fever, rash, polyarthritis, chill	-/-/-/-/-/-	Arthritis /articular fluid	-/-/-	Gas chromatography	Ankle, elbow	-/-/-/-/-/-/-	-/Penicillin G	Cure	[48, 56]
58/M	1991	France	-	-/-	Fever, polyarthritis	-/-/-/-/-/-	Septic arthritis/-	-/-/-	-	Wrist, elbow	-/-/-/-/-/-/-	-/ Cefotaxime	-	[48, 57]

Continued on next page

Patient age/Gender	Year of publication	country	Potential contact with rats or job	Bite site/ Rodent type	Clinical signs	RBC, HB, HCT%, WBC count per uL/Crp per mg/L /ESR mm/h	Complications/Isolated from	Treponemal test/RF/ANA	Identification method	Affected joints	ALT(IU/L)/AST (U/L) /UREA/CREATININ/GGT (U/L) /LIPAS/AMILASE	Antibiogram method/ Antibiotic treatment	Outcome	Ref
46/M	1992	USA	-	Finger/ Rat	Fever, chill, headache, polyarticular arthritis, pain in the lower back, elbow, and shoulder, systolic murmur, anemia	-/10/-/8200/-/-	Endocarditis /Blood culture	-/-/-	-	Wrist, sternoclavicular	-/-/-/-/-/-/-	Broth macrodilution/ Vancomycin and ceftriaxone, followed by penicillin G and tetracycline	Cure	[28]
10/F	1992	Greece	Living in a rural area	Hand/Rat	Swelling, malaise, erythematous rash, lymphadenopathy	-/14.1/43/14500/-/80	-/Blood culture	-/-/-	Phenotypic characterization	Wrist	-/-/-/-/-/-/-	-/Erythromycin	Cure	[58]
5/F	1992	Norway	-	Contact with dead pet rat without history of biting	Fever, headache, nausea, maculopapular and petechial rash	-/-/-12000/< 11/-	Septicemia/ Blood culture	-/-/-	Phenotypic characterization, gas chromatography	-	-/-/-/-/-/-/-	Disk diffusion/Penicillin and chloramphenicol	Cure	[59]
54/F	1992	Spain	Rural environment	Her lower right limb/Rat	Nausea, fever, swelling	-/-/-/-/-/-	-/Joint fluid	+/-/-	Phenotypic characterization and cellular fatty acid patterns	Ankle, wrist, elbow, knee	-/-/-/-/-/-/-	Disk diffusion and micro broth dilution/ Penicillin G	Cure	[60]

Continued on next page

Patient age/Gender	Year of publication	country	Potential contact with rats or job	Bite site/ Rodent type	Clinical signs	RBC, HB, HCT%, WBC count per uL/Crp per mg/L /ESR mm/h	Complications/Isolated from	Treponemal test/RF/ANA	Identification method	Affected joints	ALT(IU/L)/AST (U/L) /UREA/CREATININ/GGT (U/L) /LIPAS/AMILASE	Antibiogram method/ Antibiotic treatment	Outcome	Ref
19/F	1993	France	-	Patient has one dwarf rabbit, one hamster, and one pet rat that they are nibbled her playfully	Fever, polyarthralgia, rash, swelling, arthritis	-/-/-/11000/-/120	Cutaneous abscess/ Blood culture	-/-/-	-	Knee, shoulder	-/-/-/-/-/-/-	-/Amoxicillin-clavulanate	Cure	[61]
63/M	1992	USA	Pig farmer	Could not recall a rat bite or contact with rodents	Malaise, anorexia, swelling, petechial rash, fever	-/12/-/18500/-/-	Septic arthritis/Synovial fluid	-/-/-	-	Ankles, knees, wrist	48/-/-/-/134/	Disk diffusion/Flucloxacillin, followed by vancomycin	Cure	[62]
22 months/F	1994	USA	-	Contact with pet rat [died]	Fever, maculopapular rash, arthritis, arthralgia	-/-/-/-/-/-	-	-/-/-	-	-	-/-/-/-/-/-/-	-/ Nafcillin and penicillin	Cure	[41, 63]
26/F	1996	USA	-	-/-	Fever, burning left-lower-quadrant pain, chill	-/-/-/-/-/-	Female genital tract infection/ Ovarian abscess and intrauterine device	-/-/-	Phenotypic characterization, gas chromatography	-	-/-/-/-/-/-/-	-/Tobramycin and cefoxitin, followed by doxycycline	Cure	[64]

Continued on next page

Patient age/Gender	Year of publication	country	Potential contact with rats or job	Bite site/ Rodent type	Clinical signs	RBC, HB, HCT%, WBC count per uL/Crp per mg/L /ESR mm/h	Complications/Isolated from	Treponemal test/RF/ANA	Identification method	Affected joints	ALT(IU/L)/AST (U/L) /UREA/CREATININ/GGT (U/L) /LIPAS/AMILASE	Antibiogram method/ Antibiotic treatment	Outcome	Ref
22/F	1996	USA	-	-/-	Vaginal pain	-/-/-/-/-/-	Female genital tract infection/ Abscess material	-/-/-	Phenotypic characterization, gas chromatography	-	-/-/-/-/-/-/-	-/Metronidazole and clotrimazole	Cure	[64]
1-week old/F	1996	USA	-	None	Presence nodule on the her head	-/-/-/-/-/-	Nodule/ Purulent material extracted from nodule	-/-/-	Phenotypic characterization, gas chromatography	-	-/-/-/-/-/-/-	-/Cephalexin	Cure	[64]
77/M	1996	Spain	-	-/Rat	Fever, malaise, vomiting, polyarthritis	-/-/-/-/-/-	Septic oligoarthritis/-	-/-/-	-	Shoulder	-/-/-/-/-/-/-	-/Clindamycin and ciprofloxacin	-	[48,65]
9/F	1998	USA	Traveled to rural Wisconsin	Heels, hands, elbows / Pet rat	Nausea, vomiting, fever, arthralgia, fatigue, hemorrhagic pustules, anemia, malaise, arthralgia	-/11.6/-/8500/-/49	-/Blood culture	-/-/-	Phenotypic characterization, gas chromatography	Knee, elbow, ankle	-/-/-/-/-/-/-	-/Ceftriaxone	Cure	[66]
37/M[*6]	2000	Switzerland	-	Finger/ Pet rat	Arthralgia, swelling, fever, chill, pustular rash, anemia, holosystolic murmur, vegetation on the mitral valve	-/9.3/-/6700/36/150	Endocarditis / Blood culture	-/-/-	Phenotypic characterization, gas chromatography	Ankle, knee, elbow	41/65/-/-/-/-/-	Micro broth dilution/Ceftriaxone, followed by ceftriaxone and gentamicin, followed by penicillin	Cure	[67]

Continued on next page

Patient age/Gender	Year of publication	country	Potential contact with rats or job	Bite site/ Rodent type	Clinical signs	RBC, HB, HCT%, WBC count per uL/Crp per mg/L /ESR mm/h	Complications/Isolated from	Treponemal test/RF/ANA	Identification method	Affected joints	ALT(IU/L)/AST (U/L) /UREA/CREATININ/GGT (U/L) /LIPAS/AMILASE	Antibiogram method/ Antibiotic treatment	Outcome	Ref
12/M	2000	USA	-	-/Rat	Pharyngitis [sore throat], fever, hip arthritis, erythematous maculopapular rash	-/-/-/-/-/-	Septic arthritis/-	-/-/-	-	Hip	-/-/-/-/-/-/-	-/Cefuroxime and ceftriaxone	-	[48, 68]
16/M	2001	Belgium	-	Finger/ Rat	Headache, vomiting, fever	-/-/-/26300/22.2/-	-/Blood culture	-/-/-	Phenotypic characterization, gas chromatography	-	-/-/-/-/-/-/-	Disk diffusion/Amoxicillin-clavulanate	Cure	[69]
13/F	2001	UK	-	Finger/ Pet rat	Malaise, arthralgia, fever	-/-/-/NO/ 69/100	Septic arthritis/ Seropurulent material	-/-/-	-	Hip	-/-/-/-/-/-/-	-/Penicillin G, followed by amoxycillin	Cure	[49,70]
84/F	2001	Spain	Living in a rural community	Foot/Rat	Polyarthritis, swelling, erythematous rash	-/-/-/15000/206/67	Synovitis with pitting edema/ Blood culture	-/N/N	Phenotypic characterization	hands, knees, feet	NO/NO/NO/NO/-/-/-	-/Amoxicillin-clavulanate	Cure	[71]
19/F	2001	UK	-	-/Pet rat	Fever, nausea, lethargy, tiredness, haemorrhagic pustules formation, swelling	-/-/-/-/abnormal/abnormal	-/Blood culture was negative	-/-/-	Direct microscopy on pustule aspiration	Knees, ankles, metacarpophalangeal	-/-/-/-/-/-/-	-/Penicillin G	Cure	[72]
16/M	2001	USA	-	-/Rat	Polyarthritis, fever, sore throat	-/-/-/-/-/-	Septic arthritis/-	-/-/-	-	Ankle, knee, shoulder	-/-/-/-/-/-/-	-/Penicillin G	-	[48,73]
8/F	2002	USA	-	Allowed her pet rat to lick her tongue	Fever, maculopapular and petechial rash, abdominal pain, swelling, thrombocytopenia	-/-/33/10000/-/62	-/Skin aspirate	-/-/-	-	Knee, wrists	-/-/-/-/-/-/-	-/Ceftriaxone and doxycycline	Cure	[41]

Continued on next page

Patient age/Gender	Year of publication	country	Potential contact with rats or job	Bite site/ Rodent type	Clinical signs	RBC, HB, HCT%, WBC count per uL/Crp per mg/L /ESR mm/h	Complications/Isolated from	Treponemal test/RF/ANA	Identification method	Affected joints	ALT(IU/L)/AST (U/L) /UREA/CREATININ/GGT (U/L) /LIPAS/AMILASE	Antibiogram method/ Antibiotic treatment	Outcome	Ref
19/F	2002	USA	Student	-/Rat	Fever, petechial rash, bilateral ankle and shoulder pain, swelling	NO/-/-/NO/-/NO	Leukocytoclastic vasculitis /Blood culture	-/N/N	-	Hands, shoulders, ankles	-/-/-/-/-/-	-/Ampicillin –sulbactam	Cure	[74]
87/M	2003	Spain	Live in an urban area with a dog and a cat as pets.	Could not recall a rat bite or contact with rodents	Fever, weight loss, dysuria	-/-/-/-/-/-	Bacteremia/ Blood culture	-/-/-	Phenotypic characterization, gas chromatography	-	-/-/-/-/-/-	Disk diffusion/Levofloxacin	Cure	[75]
60/F*6	2003	USA	-	-/-	-	-/-/-/-/-/-	Endocarditis /Eye infection	-/-/-	-	-	-/-/-/-/-/-	-/-	Cure	[76]
72/M*13	2003	Switzerland	Living in an urban	Finger/ Rat	Polyarthritis, swelling	-/-/-/11300/120/125	Rheumatoid arthritis /Synovial fluid	-/-/-	16S rRNA gene sequencing	Knee, elbows, metacarpophalangeal	-/-/-/-/-/-	-/Penicillin G	Cure	[77]
56/F	2003	France	-	Finger/ Pet rat	Swelling, polyarthritis	-/-/-/13700/105/63	Arthritis /Blood culture	N/N/N	Phenotypic characterization and 16S rRNA gene sequencing	Knee, ankle, shoulder, elbow, wrist	-/-/-/-/-/-	Disk diffusion/Penicillin G and ofloxacin	Cure	[49, 78]

Continued on next page

Patient age/Gender	Year of publication	country	Potential contact with rats or job	Bite site/ Rodent type	Clinical signs	RBC, HB, HCT%, WBC count per uL/Crp per mg/L /ESR mm/h	Complications/Isolated from	Treponemal test/RF/ANA	Identification method	Affected joints	ALT(IU/L)/AST (U/L) /UREA/CREATININ/GGT (U/L) /LIPAS/AMILASE	Antibiogram method/ Antibiotic treatment	Outcome	Ref
62/M	2003	Singapore	-	Foot/Rat	Fever, polyarthritis, cholestatic hepatitis, swelling	-/12.5/-/29300/197/-	Septic arthritis/ Synovial fluid	-/-/-	-	Ankle, shoulder ,elbow, wrist, knee, interphalangeal, metacarpophalangeal, midtarsal	230/63/N0/NO/-/-/-	-/Ciprofloxacin and doxycycline , followed by penicillin G	Cure	[48,79]
56/M	2003	UK	-	Hand/ Rat	Fevers, cough, sore throat, diarrhea, polyarthritis, maculopapular rash, erythematous synovitis, anemia	-/11.2/-/12600/225/79	Systemic vasculitis/Joint fluid	-/-/-	DNA sequencing	Wrist, thumb, both feet, ankle	-/-/17.7/169/-/-/-	-/Penicillin and flucloxacillin, followed by doxycycline	Cure	[80]
8/F	2004	USA	-	She bought a mouse from a pet store, but it died on the first day of the patient's illness.	Fever, vomiting, sore throat, headache, papules and petechial rash, myalgia, neck pain, anemia	-/11/35/8800/-/-	-/-	-/-/-	-	-	-/-/-/-/-/-/-	-/Penicillin G	Cure	[81]

Continued on next page

Patient age/Gender	Year of publication	country	Potential contact with rats or job	Bite site/ Rodent type	Clinical signs	RBC, HB, HCT%, WBC count per uL/Crp per mg/L /ESR mm/h	Complications/Isolated from	Treponemal test/RF/ANA	Identification method	Affected joints	ALT(IU/L)/AST (U/L) /UREA/CREATININ/GGT (U/L) /LIPAS/AMILASE	Antibiogram method/ Antibiotic treatment	Outcome	Ref
11/F	2004	USA	-	-/-	Arthralgia, headache, anorexia, fatigue, myalgia, fever, petechial rash, vomiting, abdominal pain	-/13.8/39/15600/-/-	-/-	-/-/-	-	Elbow, knee	-/-/-/-/-/-/-	-/Penicillin G	Cure	[81]
13/F	2004	USA	Student	Nose and finger/Rat and hamster	Headache, fever, pustular and maculopapular rash, myalgia, vomiting, anemia	-/11.8/34.4/7100/-/-	-/-	N/-/-	-	Wrist	-/-/NO/NO/-/-/-	-/Penicillin G	Cure	[81]
23/F	2005	Netherland	Assistant at a veterinary clinic	Contact with pet rat without biting	Fever, arthralgia, maculopapular rash, swelling	-/-/-/-/22/-	-/ Blood culture	-/-/N	PCR-PCR was positive for *S. moniliformis* in the saliva of the patient's rats	Knees, wrists	-/-/-/-/-/-/-	-/Clarithromycin	Cure	[82]
7/M	2005	France	Eating pet feces	Contact with pet rat without biting	Fever, maculopapular rash, arthralgia, swelling	-/-/-/NO/300/60	-/ Blister fluid	-/-/-	Phenotypic characterization, 16S rRNA gene sequencing	ankles, knees, elbows, wrists	-/-/-/-/-/-/-	Unsuccessful/ Erythromycin and amoxicillin	Cure	[83]

Continued on next page

Patient age/Gender	Year of publication	country	Potential contact with rats or job	Bite site/ Rodent type	Clinical signs	RBC, HB, HCT%, WBC count per uL/Crp per mg/L /ESR mm/h	Complications/Isolated from	Treponemal test/RF/ANA	Identification method	Affected joints	ALT(IU/L)/AST (U/L) /UREA/CREATININ/GGT (U/L) /LIPAS/AMILASE	Antibiogram method/ Antibiotic treatment	Outcome	Ref
60/F	2005	France	Pet shop employee	Hand/ Rat	Arthralgia, swelling, fever	-/-/-/20000/105/63	Septic arthritis/Joint fluid	-/N/N	16S rRNA gene sequencing	Hands, ankles, Knees, elbows	90/40/NO/NO/-/-/-	-/Penicillin, followed by rifampin and clindamycin	Cure	[48, 84]
52/F	2005	USA	Pet shop employee	Finger/ Rat	Headache, abdominal pain, diarrhea, lethargy, lymphadenopathy, myalgia, arthralgia, anemia, maculopapular rash, thrombocytopenia	-/8.6/-/13800/-/-	Multiple organ failure /Blood culture	-/-/-	16S rRNA gene sequencing	-	112/154/55/2.9/-/-/-	-/ Ciprofloxacin, metronidazole, and vancomycin	Died	[85]
61/F	2006	Thailand	Retired nurse	Thumb / Rodent	Fever, myalgia, arthritis, chill, petechial rash, swelling, pansystolic murmur, thrombocytopenia	-/-/-/10000/-/93	Septic arthritis/-	N/N/N	-	Fingers, knees, wrists, interphalangeal	-/-/-/-/-/-/-	-/Amoxicillin/clavulanic acid and doxycycline, followed by ceftriaxone and penicillin G	Cure	[86]
68/M*6	2006	Canada	Farmer	Could not recall a rat bite	Polyarthritis, petechial rash, swelling	-/-/-/19250/-/-	Haverhill Fever, Meningitis/ Blood culture	-/-/-	-	Knees, ankles, metacarpophalangeal, wrists, elbows, shoulders	-/-/-/-/-/-/-	-/Penicillin G	Cure	[87]

Continued on next page

Patient age/Gender	Year of publication	country	Potential contact with rats or job	Bite site/ Rodent type	Clinical signs	RBC, HB, HCT%, WBC count per uL/Crp per mg/L /ESR mm/h	Complications/Isolated from	Treponemal test/RF/ANA	Identification method	Affected joints	ALT(IU/L)/AST (U/L) /UREA/CREATININ/GGT (U/L) /LIPAS/AMILASE	Antibiogram method/ Antibiotic treatment	Outcome	Ref
18/M	2006	India	-	-/Rat	Fever, cough, epistaxis, palpitations, joint pain, vegetations on the septal leaflet of the tricuspid valve, ventricular septal defect, thrombocytopenia	-/-/-/7600/-/70	Endocarditis / Blood culture	-/-/-	Phenotypic characterization	-	-/-/-/-/-/-/-	Disk diffusion/Penicillin G and gentamicin	Cure	[88]
49/F	2006	Australia	-	Hand/ Rat	Fever, joint pain, myalgia, fatigue, swelling, macular rash, tachycardia, anemia	-/10.9/-/15000/502/55	Septic arthritis/Joint fluid	N/N/N	Phenotypic characterization and 16S rRNA gene sequencing	Hands, elbow, knees, metacarpophalangeal, wrists, interphalangeal	NO/NO/NO/NO/NO/-/-	-/Doxycycline	Cure	[48]
14/M	2006	Canada	-	Caring for 2 pet rats and could not recall a rat bite	Fever, polyarthritis, petechial and purpuric rash	-/-/-/-/-/-	Leukocytoclastic vasculitis /Pustular material	-/-/-	-	Shoulders, wrists, Knees, ankles	-/-/-/-/-/-/-	-/Penicillin G	Cure	[89]
26/F	2006	USA	-	Patient let her pet rats lick her teeth	Arthralgia, chill, fever, vomiting, myalgia, petechial and purpuric rash	-/-/-/-/-/-	Leukocytoclastic vasculitis / Blood culture	+/-/+	-	-	-/-/-/-/-/-/-	-/Penicillin G	Cure	[90]

Continued on next page

Patient age/Gender	Year of publication	country	Potential contact with rats or job	Bite site/ Rodent type	Clinical signs	RBC, HB, HCT%, WBC count per uL/Crp per mg/L /ESR mm/h	Complications/Isolated from	Treponemal test/RF/ANA	Identification method	Affected joints	ALT(IU/L)/AST (U/L) /UREA/CREATININ/GGT (U/L) /LIPAS/AMILASE	Antibiogram method/ Antibiotic treatment	Outcome	Ref
60/F	2007	Taiwan	-	Toe/Rodent	Fever, weakness, weight loss, anemia, posterior eccentric mitral regurgitation, thrombocytopenia	-/8.2/-/15100/162.9/14	Endocarditis / Blood culture was negative	-/-/-	16S rRNA gene sequencing on valve specimens	-	NO/NO/-/-/-/-/-	-/Levofloxacin, followed by ceftriaxon, gentamicin, and doxycycline, followed by ceftriaxone and doxycycline	Cure	[91]
58/F*13	2007	Hong Kong	House wife	Thumb/ Rat	Fever, polyarthralgia	-/11.9/-/-/38.3/-	Septic arthritis/ Blood culture	-/-/-	Phenotypic characterization	Wrists, knee	-/-/-/-/-/-/-	-/Ampicillin	Cure	[49]
19/M	2007	New Zealand	-	Finger / Pet rat	Fever, hypotension, lymphadenopathy, headache, backache	-/-/-/17000/-/-	Sepsis/ Blood culture	-/-/-	Phenotypic characterization, 16S rRNA gene sequencing	-	-/-/-/-/-/-/-	Disk diffusion and E-test/ Flucloxacillin and gentamicin, followed by amoxicillin	Cure	[92]
29/M	2007	Germany	Farmer	-/-	Fever, dyspnea, dizziness, giant floating vegetations on a degenerated, insufficient aortic valve, lethargy, weakness	-/-/-/-/-/-	Endocarditis / Blood culture	-/-/-	Phenotypic characterization, 16S rRNA gene sequencing	-	-/-/-/-/-/-/-	-/Ampicillin, ceftriaxone, gentamicin, followed by fosfomycin, penicillin, gentamicin	Cure	[93]

Continued on next page

Patient age/Gender	Year of publication	country	Potential contact with rats or job	Bite site/ Rodent type	Clinical signs	RBC, HB, HCT%, WBC count per uL/Crp per mg/L /ESR mm/h	Complications/Isolated from	Treponemal test/RF/ANA	Identification method	Affected joints	ALT(IU/L)/AST (U/L) /UREA/CREATININ/GGT (U/L) /LIPAS/AMILASE	Antibiogram method/ Antibiotic treatment	Outcome	Ref
74/F	2007	-	-	-/-	Fever, murmur	-/-/-/-/-/-	Endocarditis / Blood culture	-/-/-	-	-	-/-/-/-/-/-/-	-/Penicillin G	Cure	[37, 94]
80/M	2008	France	Farmer	Hand /Rooster scratches	Chill, fever, back pain	-/-/-/19000/488/-	Spondylodiscitis and psoas abscess/ Blood culture	-/-/-	16S rRNA gene sequecing on blood culture sample	-	-/-/-/-/-/-/-	-/Ofloxacin and amoxicillin-clavu lanic acid, followed by imipenem-cilastatin, ciprofloxacin, teicoplanin, followed by ofloxacin, clindamycin, metronidazole	Cure	[95]
74/F	2008	Japan	-	Finger/ Rat	Fever, arthralgia, muscle pain, fatigue, erythematous rash	4410000/12.5/-/11500/14.2/-	-/Blood and urine cultures were negative	-/-/-	16S rRNA gene on crust of the bite sites	Large and small joints, and lumbar back	16/22/21/.92/-/-/220	-/Minocycline, followed by piperacillin	Cure	[96]
55/-	2009	Denmark	Farmer	Finger/ Rat	Swelling	-/-/-/-/-/-	Cutaneous abscess/ Pus	-/-/-	-	Knuckle	-/-/-/-/-/-/-	-/Erythromycin, followed by penicillin G	Cure	[97]
69/M	2009	Denmark	Farmer	Finger/ Rat	Nausea, vomiting, fever	-/-/-/NO/69/-	-/Blood culture	-/-/-	-	NO	-/-/-/-/-/-/-	-/Penicillin G	Cure	[97]

Continued on next page

Patient age/Gender	Year of publication	country	Potential contact with rats or job	Bite site/ Rodent type	Clinical signs	RBC, HB, HCT%, WBC count per uL/Crp per mg/L /ESR mm/h	Complications/Isolated from	Treponemal test/RF/ANA	Identification method	Affected joints	ALT(IU/L)/AST (U/L) /UREA/CREATININ/GGT (U/L) /LIPAS/AMILASE	Antibiogram method/ Antibiotic treatment	Outcome	Ref
13/M	2010	Canada	-	Could not recall a rat bite but kiss the pet rats	Fever, vomiting, headaches, arthralgia, myalgia, macular rash	-/12.8/-/20000/-/-	-/Blood culture	-/-/-	-	-	NO/NO/NO/NO/-/-/-	-/Penicillin G	Cure	[98]
7/F	2010	Canada	-	Could not recall a rat bite but kiss the pet rats	Fever, fatigue, arthralgia, pustules rash, swelling	-/13.3/-/14000/53.4/27	Leukocytoclastic vasculitis / Blood culture and biopsies of a pustule were negative	-/-/-	-	Wrist, ankle	-/-/-/-/-/-/-	-/Penicillin G	Cure	[98]
59/M	2010	Hawaii	-	Could not recall a rat bite or contact with rodents but observed rats around his apartment	Fever, swelling, loss of appetite, diarrhea, malaise, mitral valve abnormality	-/14.9/42.9/12400/-/-	Septic arthritis/ Synovial fluid	N/N/N	16S rRNA gene sequencing	Ankles, knees, wrists, elbow	166/229/24/1.1/-/-/-	-/Ceftriaxone, doxycycline, gentamicin, followed by penicillin, doxycycline, gentamicin	Cure	[99]

Continued on next page

Patient age/Gender	Year of publication	country	Potential contact with rats or job	Bite site/ Rodent type	Clinical signs	RBC, HB, HCT%, WBC count per uL/Crp per mg/L /ESR mm/h	Complications/Isolated from	Treponemal test/RF/ANA	Identification method	Affected joints	ALT(IU/L)/AST (U/L) /UREA/CREATININ/GGT (U/L) /LIPAS/AMILASE	Antibiogram method/ Antibiotic treatment	Outcome	Ref
8/F	2010	Canada	-	History of rat exposure	Fever, abdominal pain, nausea, diarrhea, petechial rash, headache, vomiting, malaise, arthralgia, swelling, anemia, thrombocytopenia	-/10.1/-/9600/152/45	-/Purpuric lesion biopsy	-/-/-	-	Metacarpeal	NO/NO/-/-/NO/-/-	-/Penicillin G	Cure	[98]
2 years and 3 months/F	2010	Kuwait	-	Could not recall a rat bite or contact with rodents	Cough, vomiting, seizures, pharyngitis [sore throat]	-/-/-/-/11.99/24	Bacteremia/ Blood culture	-/-/-	-	-	-/-/-/-/-/-/-	Disk diffusion and E-test/ Cefotaxime and clarithromycin	Cure	[100]
50/M	2010	Spain	-	-/Rat	Malaise, fever, diarrhea, popular and pustular rash, arthralgia, systolic murmur	-/-/-/11700/-/100	Endocarditis / Blood culture and skin biopsy	N/N/N	PCR	Interphalangeal, knee	-/-/-/-/-/-/-	-/Cefotaxime	Cure	[101]
35/M	2010	-	-	-/Rat	Vomiting, diarrhea, debilitating migratory, polyarthralgia, fever, rash	-/-/-/-/-/-	Bacteremia/ Blood culture	-/-/-	-	-	-/-/-/-/-/-/-	-/Ampicillin-sulbactam and gentamicin	Cure	[37,102]
14-month-old/M	2011	USA	-	Knee and arm/-	Fever, macular rash, hepatomegaly, lymphadenopathy, polyarthritis	-/-/-/-/-/-	-/Cerebrospinal fluid	-/-/-	-	-	-/-/-/-/-/-/-	-/-	Died	[103]
70/M	2011	Israel	-	-/-	Swellings in lateral bulbar	-/-/-/-/-/-	Polyarthralgia/-	-/-/-	16S rRNA gene sequencing	-	-/-/-/-/-/-/-	-/Ceftriaxone	Cure	[104]

Continued on next page

Patient age/Gender	Year of publication	country	Potential contact with rats or job	Bite site/ Rodent type	Clinical signs	RBC, HB, HCT%, WBC count per uL/Crp per mg/L /ESR mm/h	Complications/Isolated from	Treponemal test/RF/ANA	Identification method	Affected joints	ALT(IU/L)/AST (U/L) /UREA/CREATININ/GGT (U/L) /LIPAS/AMILASE	Antibiogram method/ Antibiotic treatment	Outcome	Ref
52/F	2011	UK	-	Finger/ Rat	Fever, pain and swelling in her finger, migrating polyarthritis, flu-like symptoms, lymphadenopathy	-/-/-/11500/24/-	Flexor tenosynovitis/ Blood culture and wound swabs were negative	-/-/-	-	-	-/-/-/-/-/-/-	-/Erythromycin, followed by flucloxacillin, benzylpenicillin and metronidazole	Cure	[105]
89/F	2011	France	-	Contact with rat	Fever	-/-/-/-/-/-	-/Blood culture	-/-/-	16S rRNA gene sequencing on blood culture	-	-/-/-/-/-/-/-	-/Amoxicillin/clavulanate and gentamicin, followed by ceftazidime, vancomycin, metronidazole, followed by rifampicin and doxycycline	Died	[106]

Continued on next page

Patient age/Gender	Year of publication	country	Potential contact with rats or job	Bite site/ Rodent type	Clinical signs	RBC, HB, HCT%, WBC count per uL/Crp per mg/L /ESR mm/h	Complications/Isolated from	Treponemal test/RF/ANA	Identification method	Affected joints	ALT(IU/L)/AST (U/L) /UREA/CREATININ/GGT (U/L) /LIPAS/AMILASE	Antibiogram method/ Antibiotic treatment	Outcome	Ref
30/M*7	2012	Australia	-	Finger/ Pet rat	Fever, headache, malaise, diarrhea, myalgia, weight loss, knee pain	-/-/-/NO/219/114	Septic arthritis/ Blood culture was negative	-/-/-	16S rRNA gene sequencing on synovial fluid	Knee	-/-/-/NO/128/-/-	-/Ceftriaxone and flucloxacilin, followed by benzylpenicillin, followed by doxycycline	Cure	[107]
8/M	2012	USA	-	Licked and scratched by pet rat	Pustular and petechiae rash, hemorrhagic pustules, swelling of the hand and digits	-/-/-/-/-/-	Arthritis/ wound	-/-/-	16S rRNA gene sequencing	joints	-/-/-/-/-/-/-	-/Penicillin G	Cure	[108]
58/M	2012	New Zealand	-	Could not recall a rat bite or contact with rodents	Fever, lower limb weakness	-/-/-/-/-/-	Spinal epidural abscess/ Abscess fluid	-/-/-	16S rRNA gene sequencing, 16S rRNA gene sequencing on epidural abscess sample	Vertebrae	-/-/-/-/-/-/-	E-test/ Ceftriaxone and ciprofloxacin, followed by ceftriaxone	Cure	[109]
28/F	2013	Canada	-	Could not recall a rat bite although hand - feeding a pet rat	Maculopapular rash, fever, pharyngitis [sore throat], oligoarthritis	-/-/-/-/-/-	-/Blood culture	-/-/-	-	-	-/-/-/-/-/-/-	-/Penicillin G	Cure	[110]

Continued on next page

Patient age/Gender	Year of publication	country	Potential contact with rats or job	Bite site/ Rodent type	Clinical signs	RBC, HB, HCT%, WBC count per uL/Crp per mg/L /ESR mm/h	Complications/Isolated from	Treponemal test/RF/ANA	Identification method	Affected joints	ALT(IU/L)/AST (U/L) /UREA/CREATININ/GGT (U/L) /LIPAS/AMILASE	Antibiogram method/ Antibiotic treatment	Outcome	Ref
44/M	2013	India	-	Finger/ Bite	Fever, mitral regurgitation	-/-/-/-/-/-	Endocarditis /Blood culture	-/-/-	Phenotypic characterization	-	-/-/-/-/-/-/-	-/Penicillin G and gentamicin	Cure	[13]
22-month-old/M	2013	USA	-	The family had two pet rats without biting	Fever, malaise, pustular rash, tachycardia, anemia	-/10.9/-/18100/5.4/ 94	Septic arthritis and possible osteomyelitis / Synovial fluid, pustule, and bone	-/-/-	16S rRNA gene sequencing and DNA mapping	Hips	-/-/-/-/-/-/-	-/Vancomycin and ceftriaxone, followed by penicillin G	Cure	[111]
3/M	2014	USA	-	Finger/Pet rat	Fever, papulovesicular rash, vomiting, leg pain, tachycardia, swelling	-/-/-/NO/15.24/-	Sepsis/synovial fluid and blood cultures were negative	-/-/-	Direct PCR/ESI-MS on synovial fluid and serum	Knee	-/-/-/-/-/-/-	-/Ampicillin, followed by amoxicillin	Cure	[112]
29/M	2014	UK	Manual labourer in a warehouse	Keeping several pet rats without biting	Fever, malaise, polyarthralgia, bilateral palmoplantar rash with vesicular, sore throat, swelling	-/-/-/10620/211/36	Septic arthritis / Blood culture was negative	-/N/N	16S rRNA gene on ankle joint fluid	Metacarpophalangeal, elbow, knee, ankles	NO/NO/NO/NO/-/-/-	-/Benzylpenicillin followed by amoxicillin	Cure	[113]

Continued on next page

Patient age/Gender	Year of publication	country	Potential contact with rats or job	Bite site/ Rodent type	Clinical signs	RBC, HB, HCT%, WBC count per uL/Crp per mg/L /ESR mm/h	Complications/Isolated from	Treponemal test/RF/ANA	Identification method	Affected joints	ALT(IU/L)/AST (U/L) /UREA/CREATININ/GGT (U/L) /LIPAS/AMILASE	Antibiogram method/ Antibiotic treatment	Outcome	Ref
49/M	2014	UK	Homeless	Could not recall a rat bite or contact with rodents	Fever, worsening right leg pain, rash, swelling, anemia, cellulitis, apical pan-systolic murmur, vegetation on the mitral valve	-/10.5/-/NO/117/-	Endocarditis / Blood culture	-/-/-	MALDI-TOF MS, 16S rRNA gene sequencing	-	-/-/-/-/-/-/-	-/Benzyl penicillin and flucloxacillin, followed by co-amoxiclav and gentamicin, followed by meropenem and doxycycline	Cure	[114]
10/M	2014	USA	-	-/Pet rat	Fever, vomiting, headache, leg pains, anemia, weakness	-/10/-/17900/-/-	-/-	-/-/-	Direct PCR on liver, lung, , and epiglottis tissue	-	-/-/-/-/-/-/-	-/-	Died	[115]
29/M	2014	UK	-	Owner of three rats	Malaise, fever, sore throat, headache, maculopapular and petechial rash, swelling	-/-/-/-/211/36	Septic arthritis and osteomyelitis/ Blood cultures and joint fluid were negative	-/N/N	16S rRNA gene sequencing on synovial fluid	Ankles, elbow, knee	-/-/-/-/-/-/-	-/Penicillin G	Cure	[116,117]

Continued on next page

Patient age/Gender	Year of publication	country	Potential contact with rats or job	Bite site/ Rodent type	Clinical signs	RBC, HB, HCT%, WBC count per uL/Crp per mg/L /ESR mm/h	Complications/Isolated from	Treponemal test/RF/ANA	Identification method	Affected joints	ALT(IU/L)/AST (U/L) /UREA/CREATININ/GGT (U/L) /LIPAS/AMILASE	Antibiogram method/ Antibiotic treatment	Outcome	Ref
19/F	2014	USA	-	Could not recall a pet rat bite or contact with rodents	Rash, swelling, fever	-/13.3/-/15800/8.5/30	Septic shoulder arthritis/Synovial fluid was negative	-/N/N	16S rRNA gene on synovial fluid	Shoulder	-/-/-/-/-/-/-	-/Vancomycin, followed by penicillin, followed by ampicillin	Cure	[118]
50/F	2015	Canada	-	-/Pet rat	Fever, maculopapular rash, polyarthritis, lateral bulbar swellings	-/-/-/-/-/-	Septic arthritis/ Synovial fluid	-/-/-	MALDI-TOF-MS	Knee	-/-/-/-/-/-/-	-/Penicillin G	Cure	[119]
45/M*6	2015	Qatar	-	Toe /Multiple rat bites	Fever, hypotension, lymphadenopathy, anemia, valvular vegetations	-/9.8/-/15300/-/-	Endocarditis / Blood culture was negative	-/-/-	-	Ankle	-/-/-/-/-/-/-	-/Cloxacillin	Cure	[120]
79/M*8	2015	Japan	Owned a bicycle shop	Could not recall a rat bite	Fatigue, joint pain, fever, diarrhea	-/-/-/24200/205/-	Bacteremia/ Blood culture	-/-/-	Phenotypic characterization, 16S rRNA gene sequencing	Elbow, hip, knee	NO/NO/NO/NO/NO/-/-	-/Meropenem	Cure	[121]

Continued on next page

Patient age/Gender	Year of publication	country	Potential contact with rats or job	Bite site/ Rodent type	Clinical signs	RBC, HB, HCT%, WBC count per uL/Crp per mg/L /ESR mm/h	Complications/Isolated from	Treponemal test/RF/ANA	Identification method	Affected joints	ALT(IU/L)/AST (U/L) /UREA/CREATININ/GGT (U/L) /LIPAS/AMILASE	Antibiogram method/ Antibiotic treatment	Outcome	Ref
17/F	2015	USA	-	Could not recall a rat bite but encountered pet rats in his house	Hip and low back pain, fever, maculopapular rash, arthritis, nausea, vomiting, swelling	-/-/-/-/1.72/46	-/ Blood culture	-/-/-	-	Sacroiliac	-/-/-/-/-/-/-	-/Ceftriaxone	Cure	[122]
9/F	2015	USA	-	Playing and kissing the pet rats	Fever, anorexia, vomiting, diarrhea, oral ulcers, joint pain, papule and pustule rash, myalgia, anemia	-/11.6/33.6/16970/146 /64	/Blood, urine, and stool cultures were negative	-/-/-	Direct PCR on skin biopsy	N	-/-/-/-/-/-/-	-/Penicillin G	Cure	[123]
46/F	2015	Canada	-	Contact to pet rat that scratch to her chest	Fever, polyarthritis, nausea, vomiting, diarrhea	-/-/-/11100/149/76	Osteomyelitis/ Blood culture	-/N/N	-	Wrists, ankles, metatarsophalangeal	114/105/-/-/-/-/-	-/Ceftriaxone	Cure	[117, 124]

Continued on next page

Patient age/Gender	Year of publication	country	Potential contact with rats or job	Bite site/ Rodent type	Clinical signs	RBC, HB, HCT%, WBC count per uL/Crp per mg/L /ESR mm/h	Complications/Isolated from	Treponemal test/RF/ANA	Identification method	Affected joints	ALT(IU/L)/AST (U/L) /UREA/CREATININ/GGT (U/L) /LIPAS/AMILASE	Antibiogram method/ Antibiotic treatment	Outcome	Ref
72/F*6	2015	Japan	-	Could not recall a rat bite or contact with rodents; Consumed water and/or food may be contaminated	Fever, chill, back pain	3670000/12.2/-/13300/26.92/-	Bacteremia and vertebral spondylodiscitis/ Blood culture	-/-/-	16S rRNA gene sequencing	-	34/45/-/1.9/239/-/-	-/Cefazolin, followed by sulbactum/ampicillin	Cure	[125]
52/M*6	2016	Japan	-	Fingers and feet/ Rat	Arthralgia, distress, swelling, back pain, fever	-/15.3/-/10300/34.6/-	Osteomyelitis/Blood culture	-/-/-	MALDI-TOF-MS, 16S rRNA gene sequencing	Knees, ankles, wrists, shoulders	NO/NO/NO/NO/-/-/-	-/Ceftriaxone, followed by penicillin G	Cure	[126]
71/M	2016	Japan	-	Could not recall a rat bite but encountered wild rats in his house	Fever, arthralgia, purpura and petechial rash	-/-/-/16480/30.04/41	leukocytoclastic vasculitis/ Blood culture	-/N/N	16S rRNA gene sequencing	Sternoclavicular, wrist, metacarpophalangeal,	-/-/40/1.16/-/-/-	-/Ceftriaxone and azithromycin, followed by levofloxacin, followed by amoxillin	Cure	[127]

Continued on next page

Patient age/Gender	Year of publication	country	Potential contact with rats or job	Bite site/ Rodent type	Clinical signs	RBC, HB, HCT%, WBC count per uL/Crp per mg/L /ESR mm/h	Complications/Isolated from	Treponemal test/RF/ANA	Identification method	Affected joints	ALT(IU/L)/AST (U/L) /UREA/CREATININ/GGT (U/L) /LIPAS/AMILASE	Antibiogram method/ Antibiotic treatment	Outcome	Ref
17/F	2016	USA	Student-Living in the farm land	Scratched by the pet rats and contact with rat oral secretions and urine	Fever, nausea, vomiting, diarrhea, abdominal pain, maculopapular rash, chill, fatigue	-/-/-/10900/1.55/46	Septic arthritis/ Blood culture	-/-/-	MALDI-TOF-MS, nucleic acid sequence	Sacroiliac	-/-/-/-/-/-/-	-/Penicillin G, followed by ceftriaxone	Cure	[128]
50/F	2016	USA	-	Could not recall a rat bite but the pet rat licked her hand	Fever, chill, purpura rash, swelling	-/-/-/-/-/-	-/-	-/-/-	Direct PCR on blood sample	Interphalangeal, metatarsophalangeal, ankle, shoulder	-/-/-/-/-/-/-	-/Ceftriaxone and doxycycline, followed by doxycycline, followed by penicillin G	Cure	[129]
19/M	2017	-	-	-/Rat	Fever, chest pain, myalgia, weight loss	-/-/-/-/-/-	Endocarditis / Blood culture was negative, operative sample was positive	-/-/-	-	-	-/-/-/-/-/-/-	-/ Penicillin and gentamicin	Cure	[37, 130]
4/F	2017	USA	-	Biting/Pet rat	Fever, arthralgia, petechial rash	-/-/-/5100/elevated/8	Septic arthritis /Joint fluid	-/-/-	-	Wrist	-/-/-/-/-/-/-	-/Vancomycin and ceftriaxone, followed by penicillin G	Cure	[131]

Continued on next page

Patient age/Gender	Year of publication	country	Potential contact with rats or job	Bite site/ Rodent type	Clinical signs	RBC, HB, HCT%, WBC count per uL/Crp per mg/L /ESR mm/h	Complications/Isolated from	Treponemal test/RF/ANA	Identification method	Affected joints	ALT(IU/L)/AST (U/L) /UREA/CREATININ/GGT (U/L) /LIPAS/AMILASE	Antibiogram method/ Antibiotic treatment	Outcome	Ref
59/M	2017	France	Snake Keeper	Bred rats for snake food and could not recall a rat bite	Fever, arthralgia, dyspnea, swelling	-/-/-/15000/125/-	Acute tetraplegia/ Culture for blood and synovial fluid were negative	-/-/-	16S rRNA gene sequencing on synovial fluid	Knees, wrist	-/-/-/-/-/-/-	-/Amoxicillin and cloxacillin	Cure	[132]
39/M	2017	Japan	Pet shop employee	Thumb/ Rat	Fever, headache, pain and stiffness in the elbow, lymphadenopathy, arthritis	-/-/-/-/-/-	Bacteremia/ Blood culture	-/-/-	MALDI-TOF-MS	Elbow	-/-/-/-/-/-/-	Disc diffusion/ Ceftriaxone and oral doxycycline, followed by ceftriaxone, followed by amoxicillin	Cure	[133]

Continued on next page

Patient age/Gender	Year of publication	country	Potential contact with rats or job	Bite site/ Rodent type	Clinical signs	RBC, HB, HCT%, WBC count per uL/Crp per mg/L /ESR mm/h	Complications/Isolated from	Treponemal test/RF/ANA	Identification method	Affected joints	ALT(IU/L)/AST (U/L) /UREA/CREATININ/GGT (U/L) /LIPAS/AMILASE	Antibiogram method/ Antibiotic treatment	Outcome	Ref
40/M	2017	Germany	-	Finger/ Pet rat	Abdominal pain, paresthesia, hypesthesia distal	-/-/-/17000/20.3/-	Spinal epidural abscess/ Abscess material	-/-/-	MALDI-TOF-MS, 16S rRNA gene sequencing on epidural abscess sample	-	-/-/-/-/-/-/-	Disc diffusion/Ceftriaxone and metronidazole, followed by penicillin G	Cure	[134]
70/M	2017	USA	-	-/-	Nausea, arthralgia, cutaneous eruption, petechial rash and hemorrhagic vesicles	-/-/-/-/-/-	Encephalopathy and bacteremia/ blood culture was negative but bacterium isolated from wrist bulla	-/-/-	-	-	-/-/-/-/-/-/-	-/Ceftriaxone, followed by vancomycin	Cure	[135]
12/M	2017	Australia	-	-/Rat	Fever, rash, arthralgia	-/-/-/13700/140.7/-	-/Blood culture	-/-/-	MALDI-TOF-MS	-	-/-/-/-/-/-/-	-/ Cefotaxime and doxycycline,	Cure	[136,137]
52/F	2018	USA	-	Could not recall a rat bite or contact with rodents	Confusion, swelling, weakness, lethargy, fever, severe mitral regurgitation	-/-/-/22600/>270/85	Mitral valve endocarditis and septic arthritis/ Synovial flui	-/-/-	MALDI-TOF-MS, 16S rRNA gene sequencing on valve tissue	Knee, ankle	-/-/-/-/-/-/-	-/Vancomycin and piperacillin-tazobactam, followed by ceftriaxone	Cure	[138]

Continued on next page

Patient age/Gender	Year of publication	country	Potential contact with rats or job	Bite site/ Rodent type	Clinical signs	RBC, HB, HCT%, WBC count per uL/Crp per mg/L /ESR mm/h	Complications/Isolated from	Treponemal test/RF/ANA	Identification method	Affected joints	ALT(IU/L)/AST (U/L) /UREA/CREATININ/GGT (U/L) /LIPAS/AMILASE	Antibiogram method/ Antibiotic treatment	Outcome	Ref
Seven month old/M*9	2018	Israel	-	Finger/ Rat	Mild shortness of breath, hepatomegaly, enlargement and decrease in function of the right ventricle, enlargement of the left pulmonary artery, aneurysm	-/-/-/NO/mild elevation/-	Endocarditis / Blood culture was negative	-/-/-	Direct PCR on necrotic tissues of the pulmonary artery	-	-/-/-/-/-/-/-	- /Ceftriaxone	Cure	[139]
61/M*11	2018	Japan	-	-/-	Fever, headache, diarrhea fatigue, rash, anemia, thrombocytopenia	2670000/8.8/25.7/7420/13.1/-	-/Blood culture	-/-/-	Phenotypic characterization, 16S rRNA gene sequencing	-	30/79/67/4.1/-/-/-	/Ceftriaxone, followed by ampicillin /sulbactam	Cure	[140]
62/F	2018	UK	-	Could not recall a rat bite; contact with the feces of rats	Lower back pain, diarrhea, vomiting, polyarthritis	-/-/-/-/218/-	Osteomyelitis/ Spinal disc and peripheral joint specimens	-/+/-	16S rRNA gene sequencing	Intervertebral disks	NO/NO/-/210/NO/-/-	-/ Penicillin G and clindamycin, followed by amoxicillin and clindamycin	Cure	[141]
33/F	2018	-	-	Rat exposure	Fever, arthralgia, myalgia, flu-like symptoms, dyspnea, murmur	-/-/-/11700/-/-	Endocarditis / Blood culture	-	-	-	-/-/-/-/-/-/-	-/ Ceftriaxone	Cure	[37,142]

Continued on next page

Patient age/Gender	Year of publication	country	Potential contact with rats or job	Bite site/ Rodent type	Clinical signs	RBC, HB, HCT%, WBC count per uL/Crp per mg/L /ESR mm/h	Complications/Isolated from	Treponemal test/RF/ANA	Identification method	Affected joints	ALT(IU/L)/AST (U/L) /UREA/CREATININ/GGT (U/L) /LIPAS/AMILASE	Antibiogram method/ Antibiotic treatment	Outcome	Ref
54/M	2019	China	-	Foot/ Rat	Fever, chill, fatigue, malaise, myalgia, mild diarrhea, purple pustular and petechial rash, thrombocytopenia	-/17/-/13100/225/-	-/Blood culture was negative	-/-/-	Meta-next generation sequencing [mNGS], 16S rRNA gene sequencing on pustular sample	-	93/-/-/-/-/-/-	-/Tazobactam/piperacillin, added doxycycline, followed by penicillin G	Cure	[143]
20/M	2019	USA	-	Could not recall a rat bite but kissed his pet rat daily	Fever, polyarthralgia, diarrhea, petechial rash, lymphadenopathy, malaise, headache, dysuria	-/13/-/11500/79.1/-	/Blood culture	-/-/-	MALDI-TOF-MS	Knees, elbows, wrists	NO/NO/-/-/-/-/-	-/Piperacillin-tazobactam, followed by ceftriaxone	Cure	[144]
24/F	2019	USA	Farmer	-/Rat	Fever, chill, vomiting, maculopapular rash, pustule formation	-/-/-/12100/-/-	-/Blood culture	N/-/-	16S rRNA gene sequencing	Knee, followed by pain in other joints	NO/NO/-/-/NO/-/-	-/Penicillin G	Cure	[145]
67/M	2019	UK	-	-/-	Ankle pain, diarrhea, purpuric rash, ejection systolic murmur, swelling	-/-/-/leukocytosis/+/-	Endocarditis / Blood culture	-/-/-	-	Metacarpal Phalangeal, sternoclavicular, ankles	-/-/-/-/-/-/-	-/Meropenem, followed by amoxicillin	Cure	[146]

Continued on next page

Patient age/Gender	Year of publication	country	Potential contact with rats or job	Bite site/ Rodent type	Clinical signs	RBC, HB, HCT%, WBC count per uL/Crp per mg/L /ESR mm/h	Complications/Isolated from	Treponemal test/RF/ANA	Identification method	Affected joints	ALT(IU/L)/AST (U/L) /UREA/CREATININ/GGT (U/L) /LIPAS/AMILASE	Antibiogram method/ Antibiotic treatment	Outcome	Ref
75/F	2019	Portugal	Living in a rural area	Finger/ Rat	Fever, myalgia, headache	-/-/-/14670/334/ -	Osteomyelitis/ Blood culture	-/-/-	16S rRNA gene sequencing, this bacterium was not identified by the phenotypic method	Sternoclavicular	NO/NO/NO/NO/-/-/-	-/Ceftriaxone, followed by amoxicillin/ clavulanate	Cure	[117,147]
36/F	2019	France	-	Hand/Pet rat	Fever, arthralgia, myalgia, headache, maculopapular rash, swelling	-/-/-/-/-/-	-/ Blood culture	-/-/-	-	Ankle, knee, wrist	-/-/-/-/-/-/-	-/Ceftriaxone, followed by amoxicillin	Cure	[148]
65/M*6	2020	Denmark	-	Toes/ Rat	Fever, myalgia, anemia, minor splenic abscess, loosening of the mitral valve prosthesis, aortic annular aneurysm, severe valve excrescences	-/7.3/-/6400/135/-	Endocarditis / Blood culture	-/-/-	MALDI-TOF-MS, whole genome-sequencing, 16S rRNA gene sequencing on heart valves	-	-/-/-/141/-/-/-	E-test/ Cefuroxime, followed by meropenem, followed by gentamicin and meropenem, and treatment was switched to benzylpenicillin	Died	[149]
24/F	2020	USA	-	-/ Pet rat	Fever, leg pain, murmur at the left sternal border	-/-/-/NO/-/-	Endocarditis /Surgical culture	-/-/-	16S rRNA gene sequencing	-	-/-/-/-/-/-/-	-/Penicillin G	Cure	[150]

Continued on next page

Patient age/Gender	Year of publication	country	Potential contact with rats or job	Bite site/ Rodent type	Clinical signs	RBC, HB, HCT%, WBC count per uL/Crp per mg/L /ESR mm/h	Complications/Isolated from	Treponemal test/RF/ANA	Identification method	Affected joints	ALT(IU/L)/AST (U/L) /UREA/CREATININ/GGT (U/L) /LIPAS/AMILASE	Antibiogram method/ Antibiotic treatment	Outcome	Ref
27/F•	2020	USA	-	Thumb/ Pet rat	Fever, chill, nausea/vomiting, malaise, polyarthralgia, erythematous and pustular rash, low back pain	-/-/-/14000/277.8/-	-/Blood culture	-/+/-	-	Hip, metacarpophalangeal, wrist, shoulder, ankle	-/-/-/-/-/-/-	-/Ceftriaxon, followed by amoxicillin/ clavulanate	Cure	[151]
71/M	2020	UK	Bus driver	-/-	Weight loss, swelling of the knee, fever, tachycardia	-/-/-/33700/224/-	Septic joint /N	-/-/-	Direct PCR on knee fluid	Knee	-/-/-/-/-/-/-	-/Co-amoxiclavand amikacin, followed by amoxicillin	Cure	[152]
66/F	2020	UK	Worked in a mail distribution center	Thumb/ Rodent	Swelling	-/-/-/19200/353/-	Periprosthetic infection and followed by septic shock /-	-/-/-	16S rRNA gene sequencing on operative tissue samples	Knee, thumb	-/-/19.9/158/-/-/-	-/Vancomycin and doxycycline, followed by meropenem	Cure	[153]
24/F	2020	USA	-	Finger/ Pet rat	Fever, chill, bilateral calf pain, holosystolic murmur	-/-/-/-/-/-	Endocarditis / Blood culture	-/-/-	Histopathology from the mitral valve	-	-/-/-/-/-/-/-	-/Penicillin G	Cure	[154]
37/M	2020	Qatar	Industrial worker	Could not recall a rat bite	Fever, vomiting, acute gastroenteritis, headache, loose stools [diarrhea], abdominal cramps, macular erythematous rash, tachycardia, thrombocytopenia	-/-/-/-/355/-	Sepsis/ Blood culture	-/-/-	-	-	98/80/11/155/-/-/-	-/Penicillin G	Cure	[155]

Continued on next page

Patient age/Gender	Year of publication	country	Potential contact with rats or job	Bite site/ Rodent type	Clinical signs	RBC, HB, HCT%, WBC count per uL/Crp per mg/L /ESR mm/h	Complications/Isolated from	Treponemal test/RF/ANA	Identification method	Affected joints	ALT(IU/L)/AST (U/L) /UREA/CREATININ/GGT (U/L) /LIPAS/AMILASE	Antibiogram method/ Antibiotic treatment	Outcome	Ref
76/M*6	2020	Japan	Living in a rural area	Present rats in the kitchen due to poor hygienic condition and could not recall a biting	Consciousness, arthralgia	-/14.9/-/18690/5.5/-	Pneumonia/ Blood culture	-/-/N	16S rRNA gene sequencing	Elbow, hip	22/32/19/1.53/ NO/-/-	Micro broth dilution/Tazobactam/piperacillin	Cure	[156]
24/F	2020	USA	-	Finger/ Pet rat	Fever, leg pain, weakness, fatigue, holosystolic murmur, multiple splenic infarcts, anemia, bilateral renal infarcts, weight loss	-/-/-/NO/-/-	Endocarditis / Blood and valve cultures were negative	-/-/-	16S rRNA gene sequencing on heart valve tissue	-	-/-/-/-/-/-	-/Ceftriaxone and vancomycin, followed by addition of clindamycin to previous antibiotics, followed by penicillin G and histamine, followed by amoxicillin/ clavulanate	Cure	[157]

Continued on next page

Patient age/Gender	Year of publication	country	Potential contact with rats or job	Bite site/ Rodent type	Clinical signs	RBC, HB, HCT%, WBC count per uL/Crp per mg/L /ESR mm/h	Complications/Isolated from	Treponemal test/RF/ANA	Identification method	Affected joints	ALT(IU/L)/AST (U/L) /UREA/CREATININ/GGT (U/L) /LIPAS/AMILASE	Antibiogram method/ Antibiotic treatment	Outcome	Ref
8-month-old/F	2021	Greece	-	Right cheek, eyes, nose, and other smaller bites all over her body /Rodent	Hypovolemic shock due to bleeding from her right cheek and nose	-/-/-/-/-/-	-/Cultures were negative	-/-/-	-	-	-/-/-/-/-/-/-	-/Ceftriaxone	Cure	[158]
8/M	2021	Australia	-	Purchased a pet rat but could not recall a rat bite	Fever, popular and petechial rash, migratory arthralgia	-/-/-/-/ 274/45	-/Blood culture	-/-/-	-	Wrist, shoulder, ankles	-/-/-/-/-/-/-	-/Ceftriaxone and doxycycline	Cure	[159]
35/M	2021	USA	-	Could not recall a rat bite but exposed to the oral flora in rats	Nausea, vomiting, fever, chills, black stools, petechial rash, migratory polymyalgias	-/-/-/9100/344.3/-	Bacteremia and synovitis /Blood culture	-/-/-	-	Ankle	-/-/-/-/-/-/-	-/Ceftriaxone	Cure	[160]

Continued on next page

Patient age/Gender	Year of publication	country	Potential contact with rats or job	Bite site/ Rodent type	Clinical signs	RBC, HB, HCT%, WBC count per uL/Crp per mg/L /ESR mm/h	Complications/Isolated from	Treponemal test/RF/ANA	Identification method	Affected joints	ALT(IU/L)/AST (U/L) /UREA/CREATININ/GGT (U/L) /LIPAS/AMILASE	Antibiogram method/ Antibiotic treatment	Outcome	Ref
55/F*10	2021	Germany	-	Contact with rat fecal when cleaning the holding cage	Fever, polyarthralgia, erythematous macules rash	-/-/-/-/5.4/-	-/Blood culture	-/NO/NO	MALDI-TOF-MS	-	35/-/-/-/52/-/-	E-test and disk diffusion/Cefuroxime, followed by penicillin G	Cure	[137]
64/M	2021	France	homeless	Forehead/ Rat	Cerebral empyema, confusion, hemiparesis, subdural hematoma	-/-/-/15082/33/-	Subdural empyema/ Empyema intraoperative sample	-/-/-	MALDI-TOF MS, 16S RNA gene sequencing	NO	-/-/-/-/-/-/-	Disk diffusion/Cefotaxim and metronidazole, followed by amoxicillin	Cure	[161]
59/F	2021	USA	Cleaning	Cleaning the place of rats in a research laboratory and could not recall a rat bite	Arthritis, swelling	-/-/-/-/-/-	Septic Polyarthritis / Synovial fluid	-/-/-	MALDI-TOF MS	Knee, wrists, shoulder, ankle	-/-/-/-/-/-/-	-/Ceftriaxone [Patient had a severe allergy to penicillin]	Cure	[162]
55/M	2021	USA	-	-/Rat	Back pain, weight loss	-/-/-/-/30.1/36	Osteomyelitis and discitis/ Aspiration of the L2-L3 disc	-/-/-	MALDI-TOF MS	-	-/-/-/-/-/-/-	Micro broth dilution/Cephalexin and linezolid, followed by cephalexin	Cure	[117]

Continued on next page

Patient age/Gender	Year of publication	country	Potential contact with rats or job	Bite site/ Rodent type	Clinical signs	RBC, HB, HCT%, WBC count per uL/Crp per mg/L /ESR mm/h	Complications/Isolated from	Treponemal test/RF/ANA	Identification method	Affected joints	ALT(IU/L)/AST (U/L) /UREA/CREATININ/GGT (U/L) /LIPAS/AMILASE	Antibiogram method/ Antibiotic treatment	Outcome	Ref
55/M	2022	Taiwan	Business man	Finger/ Rodent	Swelling, erythema	-/-/-/11600/23.40/64	Lower limb abscess formation /Blood culture	-/-/-	MALDI-TOF MS	Finger, knee	-/-/-/-/-/-/-	-/Ceftriaxone, doxycycline, and clindamycin	Cure	[163]
77/F	2022	China	Farmer	Could not recall a rat bite or contact with rodents	Fever, swelling	-/12.8/-/13100/17.83/-	Bacteremia/ Blood culture	-/N/-	MALDI-TOF MS	Shoulder	109/-/-/60.3/-/-/-	-/Ceftriaxone, followed by penicillin G	Cure	[164]
12/F	2022	Australia	-	Hands/ Kissing her pet Rodents [mice and rats] and scratches	Fever, left shoulder pain, rash, lymphadenopathy, swelling	-/-/-/-/-/-	-/Blood culture and synovial fluid	-/-/-	-	Glenohumeral	-/-/-/-/-/-/-	-/Ceftriaxone, followed by amoxycillin	Cure	[165]
45/M*7	2022	USA	-	Hands/ Rat	Fever, arthralgia, headache, weight loss, myalgia, abdominal pain	-/17.5/-/15000/118/-	HIV/Joint fluid	N/-/-	MALDI-TOF MS	Knee, wrist	-/63/-/-/-/-/-	-/Ceftriaxone	Cure	[166]

Continued on next page

Patient age/Gender	Year of publication	country	Potential contact with rats or job	Bite site/ Rodent type	Clinical signs	RBC, HB, HCT%, WBC count per uL/Crp per mg/L /ESR mm/h	Complications/Isolated from	Treponemal test/RF/ANA	Identification method	Affected joints	ALT(IU/L)/AST (U/L) /UREA/CREATININ/GGT (U/L) /LIPAS/AMILASE	Antibiogram method/ Antibiotic treatment	Outcome	Ref
64/F*6	2022	Netherland	-	Could not recall a rat bite or contact with rodents	Fever, swelling	-/-/-/13100/156/-	Periprosthetic joint infection and bacteremia/ Blood culture and knee aspirate	-/-/-	16S rRNA gene sequencing on colonial bacterium, 16S rRNA gene sequencing on knee aspirate	Knee	-/-/-/-/-/-/-	-/Cefuroxime, followed by ceftriaxone, followed by doxycycline	Cure	[167]
44/M	2022			-/Rat	Lethargy, arthralgia, fevers, rigors	-/-/-/-/-/-	Endocarditis /Blood culture	-/-/-	-	-	-/-/-/-/-/-/-	-/-	Cure	[37, 168]
91/M	2023	Belgium	-	Hand/ Rat	Swelling, cellulitis with subcutaneous abscess, septic mono-arthritis, erythema	-/-/-/23750/158/-	Thumb abscess/ Abscess material	-/-/-	MALDI-TOF MS	Wrist, thumb	-/-/-/-/-/-/-	E-test/ Amoxicillin /clavulanate, followed by doxycycline	Cure	[169]
34/F	2023	USA	-	-/Rat	Fever, malaise, chill, polyarthralgia erythematous vesicular and papular rash, anemia	-/11.9/-/9400/-/-	Septic vasculitis/N	N/-/-	16S rRNA gene sequencing on tissue and immunohistochemistry	-	-/-/-/-/-/-/-	-/Ceftriaxone, followed by doxycycline	Cure	[170]

Continued on next page

Patient age/Gender	Year of publication	country	Potential contact with rats or job	Bite site/ Rodent type	Clinical signs	RBC, HB, HCT%, WBC count per uL/Crp per mg/L /ESR mm/h	Complications/Isolated from	Treponemal test/RF/ANA	Identification method	Affected joints	ALT(IU/L)/AST (U/L) /UREA/CREATININ/GGT (U/L) /LIPAS/AMILASE	Antibiogram method/ Antibiotic treatment	Outcome	Ref
51/M	2023	Poland	Homeless	The patient's previous job was exterminating rodents	Fever, palpable purpura rash, arthralgia	-/-/-/20500/265.7/-	-/Blood culture	-/N/N	-	Wrists, ankles	32/58/-/1.4/188/283/292	-/Ceftriaxone and vancomycin, added ampicillin	Cure	[171]
75/F	2023	USA	-	Finger/Pet rat	Back pain, fever, chill, anemia, thickening of the posterior mitral leaflet, mitral regurgitation	-/10.1/-/11800/-/-	Endocarditis/Blood culture	-/-/-	-	-	-/-/15/.5/-/-/-	-/Ceftriaxone and azithromycin, followed by ceftriaxone	Cure	[37]
59/F*14	2023	USA	-	Finger/Pet rat	Swelling, fever, chill	-/-/-/NO/171/110	Acute Polyarthritis/Synovial fluid	N/N/+	-	Knees, wrist	-/-/-/-/-/-/-	Ceftriaxone	Cure	[172]

*Underlying diseases: 1: Rheumatic fever; 2: Premature boy; 3: sickle cell anemia;4: Arteriosclerotic heart disease;5: Osteoarthritis and Paget's disease;6: Diabetic; 7: HIV;8: Chronic heart failure, lumbar canal stenosis, and hepatic cell carcinoma;9: Tetralogy of Fallot;10: Atrial fibrillation hypothyroidism;11: Cirrhotic;12: Alcoholism, pulmonary tuberculosis, chronic seizure disorder;13: Osteomyelitis; 14: Autoimmune hepatitis and vitiligo; ●: Positive for anti-cyclic citrullinated peptide; M: Male, F: Female, N: Negative, NO: Normal, CRP: C-reactive protein, ESR: Erythrocyte sedimentation rate, WBC: White blood cell, VDRL: Venereal disease research laboratory, RF: Rheumatoid factor, ANA: Antinuclear antibodies, HB: Hemoglobin, HCT: Hematocrit, RBC: Red blood cell, ALT: Alanine aminotransferase, AST: Aspartate aminotransferase, GGT: Gamma-glutamyl transpeptidase, -: Not stated or incomplete description or no data

Table S2. Summary of published case reports on *S. notomytis*.

Patient age/Gender	Year of publication	country	Job	Bite site/ Rodent type	Clinical signs	RBC, HB, HCT, WBC count per uL/Crp per mg/L /ESR mm/h	Type of infection/Isolated from	Type of infection/Isolated from	Treponemal test/RF/ ANA	Identification method	Affected joints	ALT/AST/UREA/CREATININ/GGT/LIPAS/AMILASE	Antibiogram method/ Antibiotic treatment	Outcome	Ref
63/F	2018	Japan	-	Finger/ Rat	Fever, fatigue, nausea, diarrhea, arthralgia, skin rash, pustule formation	-/-/-/11600/27.0/99	-/Blood and skin pustule samples	-/Blood and skin pustule samples	-/-/-	16S rRNA gene sequencing	wrist, elbow, knee, ankle	60/-/-/2.49/-/-/-	Disk diffusion/ Ampicillin	Cure	[173]
94/F	2018	Japan	-	There is not animal biting	Fever, malaise, anorexia, bilateral knee pain, swelling	-/-/-/14200/19.5/-	-/Joint fluid	-/Joint fluid	-/-/-	*groEL*, *gyrB* and 16S rRNA genes sequencing on synovial fluid	Knees	-/-/-/-/-/-/-	-/Ceftriaxone, followed by ampicillin/ sulbactam, followed by minocycline	Died	[174]
67/M	2019	Japan	-	Rats may have bitten patient, but the wounds had healed	Fever, purpura rash, polyarthritis	-/-/-/-/10.22/-	Septic arthritis/ Blood culture	Septic arthritis/ Blood culture	-/N/N	MALDI-TOF MS, 16S rRNA gene sequencing	Interphalangeal, metacarpophalangeal, wrist, metatarsophalangeal	NO/NO/-/NO/-/-/-	Micro broth dilution/ Meropenem	Cure	[175]

Continued on next page

Patient age/Gender	Year of publication	country	Job	Bite site/ Rodent type	Clinical signs	RBC, HB, HCT, WBC count per uL/Crp per mg/L /ESR mm/h	Type of infection/Isolated from	Type of infection/Isolated from	Treponemal test/RF/ ANA	Identification method	Affected joints	ALT/AST/UREA/CREATININ/GGT/LIPAS/AMILASE	Antibiogram method/ Antibiotic treatment	Outcome	Ref
72/F	2021	Thailand	-	-/-	Fever, myalgia, polyarthralgia, decreasing consciousness, swelling	-/-/-/18900/211.6/-	Meningitis, septic polyarthritis and spondylodiscitis/Blood culture	Meningitis, septic polyarthritis and spondylodiscitis/Blood culture	-/-/-	16S rRNA genes sequencing on synovial fluid	Knee, hands, wrists	60/-/-/2.49/-/-/-	-/Ceftriaxone, switched to amoxicillin	Cure	[176]
70/F	2022	Japan	-	Exposure to rat feces and could not recall a rat bite	Myalgia, fever, purpura rash, back pain	-/-/-/9000/16.9/-	Bacteremia/ Blood culture	Bacteremia /Blood culture	-/-/-	MALDI-TOF MS, 16S rRNA gene sequencing	-	-/-/-/-/-/-/-	Micro broth dilution/ Ceftriaxone, followed by ampicillin, followed by amoxicillin	Cure	[177]

Table S3. Summary of published case reports on *S. felis*.

Patient age/Gender	Year of publication	country	Job	Bite site/ Rodent type	Clinical signs	RBC, HB, HCT,WBC count per uL/Crp per mg/L /ESR mm/h	Type of infection/Isolated from	Treponemal test/RF/A	Identification method	Affected joints	ALT/AST /UREA/CREATINI	Antibiogram method/ Antibiotic	Outcome	References
18/M	2021	Germany	-	The patient has a pet dog that was in regular contact with several cats	Fever, rash, musculoskeletal pain	-/-/-/15000/248/-	Polyarteritis nodosa/ Blood culture was negative	-/-/-	PCR on purple skin lesions	Knee	-/-/-/-/-/-/-	-/Amoxicillin/clavulanic acid, followed by ceftriaxone, followed by amoxicillin	Cure	[178]

References

1. Altemeier W, Snyder H, Howe G (1945) Penicillin therapy in rat bite fever. *JAMA* 127: 270–273. https://doi.org/10.1001/jama.1945.02860050018005
2. Petersen ES, Mccullough NB, Eisele CW, et al. (1950) Subacute bacterial endocarditis due to *Streptobacillus moniliformis*. *JAMA* 144: 621–622. https://doi.org/10.1001/jama.1950.62920080007006f
3. Blake FG (1916) The etiology of rat-bite fever. *J Exp Med* 23: 39–60. https://doi.org/10.1084/jem.23.1.39
4. Tileston W (1916) The etiology and treatment of rat-bite fever. *JAMA* 66: 995–998. https://doi.org/10.1001/jama.1916.02580400001001
5. Litterer W (1917) A new species of *Streptothrix* isolated in a case of rat bite fever. *JAMA* 68: 1287.
6. Dick GF, Tunnicliff R (1918) A *streptothrix* isolated from the blood of a patient bitten by a weasel (*Streptothrix putorii*). *J Infect Dis* 23: 183–187. https://doi.org/10.1086/infdis/23.2.183
7. Tunnicliff R, Mayer KM (1918) A case of rat-bite fever. *J Infect Dis* 23: 555–558. https://doi.org/10.1086/infdis/23.6.555
8. Scharles F, Seastone C (1934) Haverhill fever following rat-bite. *N Engl J Med* 211: 711–714. https://doi.org/10.1056/NEJM193410182111603
9. Stuart-Harris C, Wells A, Rosher A, et al. (1935) Four cases of infective endocarditis due to organisms similar to *Haemophilus pavainfluenzae*, and one case due to a pleo-morphic *Streptobacillus*. *J Pathol Bacteriol* 41: 407–421. http://dx.doi.org/10.1002/path.1700410305
10. McCormack RC, Kaye D, Hook EW (1967) Endocarditis due to *Streptobacillus moniliformis*: A report of two cases and review of the literature. *JAMA* 200: 77–79. http://dx.doi.org/10.1001/jama.1967.03120140135036
11. Dawson M, Hobby G (1939) Rat-bite fever. *Trans Assoc Am Physicians* 54: 329–332.
12. Allbritten F, Sheely R, Jeffers W (1940) Haverhillia Multiformis Septicemia: Its etiologic and clinical relationship to Haverhill and rat-bite fevers. *Journal of the American Medical Association* 114: 2360–2363. http://dx.doi.org/10.1001/jama.1940.02810240014005
13. Madhubashini M, George S, Chandrasekaran S (2013) *Streptobacillus moniliformis* endocarditis: case report and review of literature. *Indian Heart J* 65: 442–446. https://doi.org/10.1016/j.ihj.2013.06.019
14. Rountree PM, Rohan M (1941) A fatal human infection with *Streptobacillus moniliformis*. *Med J Aust* 1: 359–361. https://doi.org/10.5694/j.1326-5377.1941.tb95302.x
15. Brown TM, Nunemaker JC (1942) Rat-bite fever. A review of the american cases with revaluation of etiology; report of cases. *Bulletin of the Johns Hopkins Hospital* 70: 201–327.
16. Rosen E, Denzer BS (1944) Rat bite fever caused by *Streptobacillus moniliformis*: Case report. *J Pediatr* 24: 544–552. https://doi.org/10.1016/S0022-3476(44)80032-4
17. Kane F (1944) Rat bite fever due to *Streptobacillus moniliformis*: a case treated by penicillin. *Ulster Med J* 13: 129. https://doi.org/10.1016/s0140-6736(00)74271-1
18. Blake FG, Horstmann DM, Arnold H (1944) A fatal case of rat-bite fever due to *Streptobacillus moniliformis*. *Yale J Biol Med* 16: 589.

19. Brooksaler F (1945) Penicillin therapy in ratbite fever. *J Pediatr* 27: 442–446. https://doi.org/10.1001/jama.1945.02860050018005
20. Mcdermott W, Leask M, Benoit M (1945) *Streptobacillus moniliformis* as a cause of subacute bacterial endocarditis: report of a case treated with penicillin. *Ann Intern Med* 23: 414–423.
21. Wheeler WE (1945) Treatment of the rat bite fevers with penicillin. *Am J Dis Child* 69: 215–220. https://doi.org/10.1001/archpedi.1945.02020160015003
22. Watkins CG (1946) Ratbite fever. *J Pediatr* 28: 429–448. https://doi.org/10.1016/S0022-3476(46)80025-8
23. Levine B, Civin WH (1947) *Streptobacillus moniliformis* bacteremia with minor clinical manifestations. *Arch Intern Med* 80: 53–60. https://doi.org/10.1001/archinte.1947.00220130061004
24. Sprecher MH, Copeland J (1947) Haverhill fever due to *Streptobacillus moniliformis* treated with streptomycin. *JAMA* 134: 1014–1016. https://doi.org/10.1001/jama.1947.72880290001008
25. Priest WS, Smith JM, Mcgee CJ (1947) Penicillin therapy of subacute bacterial endocarditis: A study of the end results in thirty-four cases, with particular reference to dosage, methods of administration, criteria for judging adequacy of treatment and probable reasons for failures. *Arch Intern Med* 79: 333–359. https://doi.org/10.1001/archinte.1947.00220090093006
26. Borgen L, Gaustad V (1948) Infection with *Actinomyces muris* ratti (*Streptobacillus moniliformis*) after bite of laboratory rat. *Acta Med Scand* 130: 189–198.
27. Lominski I, Henderson AS, McNee J (1948) Rat-bite fever due to *Streptobacillus moniliformis*. *Br Med J* 2: 510. https://doi.org/10.1136/bmj.2.4575.510
28. Rupp ME (1992) *Streptobacillus moniliformis* endocarditis: case report and review. *Clin Infect Dis* 14: 769–772. https://doi.org/10.1093/clinids/14.3.769
29. Prouty M, Schafer EL (1950) Periarteritis nodosa associated with ratbite fever due to *Streptobacillus moniliformis* (erythema arthriticum epidemicum). *J Pediatr* 36: 605–613. https://doi.org/10.1016/S0022-3476(50)80127-0
30. Stokes J, Gray I, Stokes E (1951) *Actinomyces muris* endocarditis treated with chloramphenicol. *Br Heart J* 13: 247. https://doi.org/10.1136/hrt.13.2.247
31. Christiansen J, Birge EA (1951) *Streptobacillus moniliformis* infection following acute appendicitis: report of a case. *Am J Clin Pathol* 21: 1062–1064. https://doi.org/10.1093/ajcp/21.11.1062
32. Hamburger M, Knowles HC (1953) *Streptobacillus moniliformis* infection complicated by acute bacterial endocarditis: Report of a case in a physician following bite of laboratory rat. *AMA Arch Intern Med* 92: 216–220. https://doi.org/10.1001/archinte.1953.00240200066008
33. Burke WA, Kwong O, Halpern R (1959) Rat bite fever due to *Streptobacillus moniliformis*—A report of two cases. *Calif Med* 91: 356–358.
34. Holden F, Mackay J (1964) Rat-bite fever-an occupational hazard. *Can Med Assoc J* 91: 78.
35. McGill R, Martin A, Edmunds P (1966) Rat-bite fever due to *Streptobacillus moniliformis*. *Br Med J* 1: 1213. https://doi.org/10.1136/bmj.1.5497.1213
36. Carbeck RB, Murphy JF, Britt EM (1967) Streptobacillary rat-bite fever with massive pericardial effusion. *JAMA* 201: 703–704. https://doi.org/10.1001/jama.1967.03130090067024

37. Mahesh A, Padrao EMH, Randhawa R, et al. (2023) A Rodential reckoning: a case report and systematic review of streptobacillary endocarditis. *Autops Case Rep* 13: e2023423. https://doi.org/10.4322/acr.2023.423
38. Lambe Jr DW, McPhedran AM, Mertz JA, et al. (1973) *Streptobacillus moniliformis* isolated from a case of Haverhill fever: biochemical characterization and inhibitory effect of sodium polyanethol sulfonate. *Am J Clin Pathol* 60: 854–860. https://doi.org/10.1093/ajcp/60.6.854
39. Chulay JD, Lankerani MR (1976) Splenic abscess: report of 10 cases and review of the literature. *Am J Med* 61: 513–522. https://doi.org/10.1016/0002-9343(76)90331-4
40. Raffin BJ, Freemark M (1979) Streptobacillary rat-bite fever: a pediatric problem. *Pediatrics* 64: 214-217.
41. Ojukwu IC, Christy C (2002) Rat-bite fever in children: case report and review. *Scand J Infect Dis* 34: 474–477. https://doi.org/10.1080/003655402320170345
42. Faro S, Walker C, Pierson RL (1980) Amnionitis with intact amniotic membranes involving *Streptobacillus moniliformis*. *Obstet Gynecol* 55: 9S–11S. https://doi.org/10.1097/00006250-198003001-00003
43. Shanson D, Midgley J, Gazzard B, et al. (1983) *Streptobacillus moniliformis* isolated from blood in four cases of Haverhill fever: first outbreak in Britain. *The Lancet* 322: 92–94. https://doi.org/10.1016/S0140-6736(83)90072-7
44. Ben-Chetrit E, Nashif M, Levo Y (1983) Infective endocarditis caused by uncommon bacteria. *Scand J Infect Dis* 15: 179–183. https://doi.org/10.3109/inf.1983.15.issue-2.09
45. Dijkamns B, Thomeer R, Vielvoye G, et al. (1984) Brain abscess due to *Streptobacillus moniliformis* and *Actinobacterium meyeri*. *Infection* 12: 262–264. https://doi.org/10.1007/bf01645956
46. McHugh TP, Bartlett RL, Raymond JI (1985) Rat bite fever: report of a fatal case. *Ann Emerg Med* 14: 1116–1118. https://doi.org/10.1016/S0196-0644(85)80936-7
47. Mandel DR (1985) Streptobacillary fever. An unusual cause of infectious arthritis. *Cleve Clin Q* 52: 203–205. https://doi.org/10.3949/ccjm.52.2.203
48. Dendle C, Woolley IJ, Korman T (2006) Rat-bite fever septic arthritis: illustrative case and literature review. *Eur J Clin Microbiol Infect Dis* 25: 791–797. https://doi.org/10.1007/s10096-006-0224-x
49. Wang TK, Wong SS (2007) *Streptobacillus moniliformis* septic arthritis: a clinical entity distinct from rat-bite fever? *BMC Infect Dis* 7: 1–7. https://doi.org/10.1186/1471-2334-7-56
50. Simon MW, Wilson HD (1986) *Streptobacillus moniliformis* endocarditis: A case report. *Clin Pediatr* 25: 110–111. https://doi.org/10.1177/000992288602500211
51. Anderson D, Marrie TJ (1987) Septic arthritis due to *Streptobacillus moniliformis*. *Arthritis Rheum* 30: 229–230. https://doi.org/10.1002/art.1780300216
52. Rumley RL, Patrone NA, White L (1987) Rat-bite fever as a cause of septic arthritis: a diagnostic dilemma. *Ann Rheum Dis* 46: 793–795. https://doi.org/10.1136/ard.46.10.793
53. Holroyd KJ, Reiner AP, Dick JD (1988) *Streptobacillus moniliformis* polyarthritis mimicking rheumatoid arthritis: an urban cae of rat bite fever. *Am J Med* 85: 711–714. https://doi.org/10.1016/S0002-9343(88)80247-X
54. Wilkins E, Millar J, Cockcroft P, et al. (1988) Rat-bite fever in a gerbil breeder. *J Infect* 16: 177–180. https://doi.org/10.1016/s0163-4453(88)94047-9

55. Sens MA, Brown EW, Wilson LR, et al. (1989) Fatal *Streptobacillus moniliformis* infection in a two-month-old infant. *Am J Clin Pathol* 91: 612–616. https://doi.org/10.1093/ajcp/91.5.612
56. Anglada A, Comas L, Euras J, et al. (1990) Arthritis caused by *Streptobacillus moniliformis*: a case of fever induced by a rat bite. *Med Clin* (Barc) 94: 535–537.
57. Ban R, Bajolet-Laudinat O, Eschard J, et al. (1991) Acute purulent polyarthritis induced by *Streptobacillus moniliformis*. *Presse Medicale* (Paris, France: 1983) 20: 1515–1516.
58. Konstantopoulos K, Skarpas P, Hitjazis F, et al. (1992) Rat bite fever in a Greek child. *Scand J Infect Dis* 24: 531–533. https://doi.org/10.3109/00365549209052640
59. Rygg M, Bruun CF (1992) Rat bite fever (*Streptobacillus moniliformis*) with septicemia in a child. *Scand J Infect Dis* 24: 535–540. https://doi.org/10.3109/00365549209052641
60. López P, Euras J, Anglada A, et al. (1992) Infection due to *Streptobacillus moniliformis*. *Clin Microbiol Newsl* 14: 38–40. https://doi.org/10.1016/0196-4399(92)90035-8
61. Vasseur E, Joly P, Nouvellon M, et al. (1993) Cutaneous abscess: a rare complication of *Streptobacillus moniliformis* infection. *Br J Dermatol* 129: 95–96. https://doi.org/10.1111/j.1365-2133.1993.tb03322.x
62. Fordham J, McKay-Ferguson E, Davies A, et al. (1992) Rat bite fever without the bite. *Ann Rheum Dis* 51: 411–412. https://doi.org/10.1136/ard.51.3.411
63. Prager L, Frenck Jr RW (1994) *Streptobacillus moniliformis* infection in a child with chickenpox. *Pediatr Infect Dis J* 13: 417–418. https://doi.org/10.1097/00006454-199405000-00019
64. Pins MR, Holden JM, Yang JM, et al. (1996) Isolation of presumptive *Streptobacillus moniliformis* from abscesses associated with the female genital tract. *Clin Infect Dis* 22: 471–476. https://doi.org/10.1093/clinids/22.3.471
65. JL AV, Trapero M (1996) *Streptobacillus moniliformis* septic oligoarthritis (fever caused by rat bite). *Rev Clin Esp* 196: 413–415.
66. Cunningham BB, Paller AS, Katz BZ (1998) Rat bite fever in a pet lover. *J Am Acad Dermatol* 38: 330–332. https://doi.org/10.1016/S0190-9622(98)70576-6
67. Rordorf T, Züger C, Zbinden R, et al. (2000) *Streptobacillus moniliformis* endocarditis in an HIV-positive patient. *Infection* 28: 393–394. https://doi.org/10.1007/s150100070012
68. Hockman DE, Pence CD, Whittler RR, et al. (2000) Septic arthritis of the hip secondary to rat bite fever: a case report. *Clin Orthop Relat Res* 380: 173–176. https://doi.org/10.1097/00003086-200011000-00023
69. Frans J, Verhaegen J, Van Noyen R (2001) *Streptobacillus moniliformis*: case report and review of the literature. *Acta Clinica Belgica* 56: 187–190. https://doi.org/10.1179/acb.2001.029
70. Downing N, Dewnany G, Radford P (2001) A rare and serious consequence of a rat bite. *Ann R Coll Surg Engl* 83: 279.
71. Torres L, Lopez A, Escobar S, et al. (2003) Bacteremia by *Streptobacillus moniliformis*: first case described in Spain. *Eur J Clin Microbiol Infect Dis* 22: 258–260. https://doi.org/10.1007/s10096-003-0891-9
72. Hudsmith L, Weston V, Szram J, et al. (2001) Rat bite fever. *Lancet Infect Dis* 1: 91. https://doi.org/10.1016/S1473-3099(01)00064-0
73. Hambridge S, Ogle J (2001) Index of suspicion. Case 1. Diagnosis: Rat-bite fever. *Pediatr Rev* 22: 95–103. https://doi.org/10.1542/pir.22-3-95

74. Gilroy SA, Khan MU (2002) Rat bite fever: case report and review of the literature. *Infect Dis Clin Pract* 11: 403–405.
75. Torres L, Lopez A, Escobar S, et al. (2003) Bacteremia by *Streptobacillus moniliformis*: first case described in Spain. European Journal of Clinical Microbiology and Infectious Diseases 22: 258–260.
76. Moshfeghi DM, Kaiser PK, Scott IU, et al. (2003) Acute endophthalmitis following intravitreal triamcinolone acetonide injection. *Am J Ophthalmol* 136: 791–796. https://doi.org/10.1016/S0002-9394(03)00483-5
77. Stehle P, Dubuis O, So A, et al. (2003) Rat bite fever without fever. *Ann Rheum Dis* 62: 894–896. https://doi.org/10.1136/ard.62.9.894
78. Wallet F, Savage C, Loïez C, et al. (2003) Molecular diagnosis of arthritis due to *Streptobacillus moniliformis*. *Diagn Microbiol Infect Dis* 47: 623–624. https://doi.org/10.1016/S0732-8893(03)00167-6
79. Thong BYH, Barkham T (2003) Suppurative polyarthritis following a rat bite. *Ann Rheum Dis* 62: 805–806. https://doi.org/10.1136/ard.62.9.805
80. Tattersall R, Bourne J (2003) Systemic vasculitis following an unreported rat bite. *Ann Rheum Dis* 62: 605–606. https://doi.org/10.1136/ard.62.7.605
81. Freels LK, Elliott SP (2004) Rat bite fever: three case reports and a literature review. *Clin Pediatr* 43: 291–295. https://doi.org/10.1177/000992280404300313
82. Van Nood E, Peters S (2005) Rat-bite fever. *Neth J Med* 63: 319–321.
83. Andre J, Freydiere A, Benito Y, et al. (2005) Rat bite fever caused by *Streptobacillus moniliformis* in a child: human infection and rat carriage diagnosed by PCR. *J Clin Pathol* 58: 1215–1216. https://doi.org/10.1136/jcp.2005.026401
84. Legout L, Senneville E, Mulleman D, et al. (2005) Rat bite fever mimicking rheumatoid arthritis. *Scand J Infect Dis* 37: 532–533. https://doi.org/10.1080/00365540510032114
85. Control CfD, Prevention (2005) Fatal rat-bite fever-Florida and Washington, 2003. M*MWR Morbidity and mortality weekly report* 53: 1198–1202.
86. Ayuthaya RKN, Niumpradit N (2005) Rat-bite fever presenting with rash and septic arthritis. *J Med Assoc Thai* 88: S247.
87. Abdulaziz H, Touchie C, Toye B, et al. (2006) Haverhill fever with spine involvement. *J Rheumatol* 33: 1409–1410.
88. Balakrishnan N, Menon T, Shanmugasundaram S, et al. (2006) *Streptobacillus moniliformis* endocarditis. *Emerg Infect Dis* 12: 1037–1038. https://doi.org/10.3201/eid1206.060069
89. Albedwawi S, LeBlanc C, Show A, et al. (2006) A teenager with fever, rash and arthritis. *CMAJ* 175: 354–354. https://doi.org/10.1503/cmaj.060309
90. Mackowiak PA, Tandon R, Lee M, et al. (2006) A 26-year-old woman with a rash on her extremities. *Clin Infect Dis* 43: 1616–1617. https://doi.org/10.1086/509585
91. Chen PL, Lee NY, Yan JJ, et al. (2007) Prosthetic valve endocarditis caused by *Streptobacillus moniliformis*: a case of rat bite fever. *J Clin Microbiol* 45: 3125–3126. https://doi.org/10.1128/jcm.01169-07
92. Sakalkale R, Mansell C, Whalley D, et al. (2007) Rat-bite fever: a cautionary tale. *N Z Med J* 120: U2545.

93. Kondruweit M, Weyand M, Mahmoud FO, et al. (2007) Fulminant endocarditis caused by *Streptobacillus moniliformis* in a young man. *J Thorac Cardiovasc Surg* 134: 1579–1580. https://doi.org/10.1016/j.jtcvs.2007.08.010
94. Forster D, Becker A, Stahlhut P, et al. (2007) P1457 *Streptobacillus moniliformis* endocarditis diagnosed by 16S rRNA gene PCR and direct sequencing applied to a resected heart valve. *Int J Antimicrob Agents* 29: S407. http://dx.doi.org/10.1016%2FS0924-8579(07)71296-7
95. Dubois D, Robin F, Bouvier D, et al. (2008) *Streptobacillus moniliformis* as the causative agent in spondylodiscitis and psoas abscess after rooster scratches. *J Clin Microbiol* 46: 2820–2821. https://doi.org/10.1128/jcm.00744-08
96. Nakagomi D, Deguchi N, Yagasaki A, et al. (2008) Rat-bite fever identified by polymerase chain reaction detection of *Streptobacillus moniliformis* DNA. *J Dermatol* 35: 667–670. https://doi.org/10.1111/j.1346-8138.2008.00541.x
97. Hagelskjaer L, Sørensen I, Randers E (1998) *Streptobacillus moniliformis* infection: 2 cases and a literature review. *Scand J Infect Dis* 30: 309–311. https://doi.org/10.1080/00365549850161016
98. Khatchadourian K, Ovetchkine P, Minodier P, et al. (2010) The rise of the rats: A growing paediatric issue. *Paediatr Child Health* 15:131–4. https://doi.org/10.1093/pch/15.3.131
99. Dworkin J, Bankowski MJ, Wenceslao SM, et al. (2010) A case of septic arthritis from rat-bite fever in Hawai ‘i. *Hawaii Med J* 69: 65.
100. Joshi RM, Al Sweih N, Bin Nakhi HA, et al. (2010) *Streptobacillus moniliformis* bacteremia in a child: case report. *Med Princ Pract* 19: 409–411. https://doi.org/10.1159/000316383
101. Barja J, Castelo L, Almagro M, et al. (2010) Rat-bite fever: A case in Spain with skin lesions. *Actas Dermosifiliogr* 101: 275–277. https://actasdermo.org/en-rat-bite-fever-a-case-in-articulo-S1578219010706326
102. Shah S, Fortuna R, Scofield S, et al. (2010) Bite the hand that fed you. Springer 233 spring ST, New York, NY 10013 USA., 496–497.
103. Banerjee P, Ali Z, Fowler DR (2011) Rat bite fever, a fatal case of *Streptobacillus moniliformis* infection in a 14-month-old boy. Journal of forensic sciences 56: 531–533.
104. Orlev A, Miskin I, Temper V, et al. (2011) A 70-year-old man with fever and polyarthralgia. *Clin Infect Dis* 53: 1037–1038. https://doi.org/10.1093/cid/cir620
105. Dewing D, Nayar R, McArthur P (2011) Rat bite fever with flexor tenosynovitis: case report and review. *Eur J Plast Surg* 34: 293–295. https://doi.org/10.1007/s00238-010-0461-1
106. Loridant S, Jaffar-Bandjee MC, La Scola B (2011) Case report: shell vial cell culture as a tool for *Streptobacillus moniliformis* “resuscitation”. *Am J Trop Med Hyg* 84: 306. https://doi.org/10.4269/ajtmh.2011.10-0466
107. Chean R, Stefanski D, Woolley I, et al. (2012) Rat bite fever as a presenting illness in a patient with AIDS. *Infection* 40: 319–321. https://doi.org/10.1007/s15010-011-0181-x
108. Islam S, Cooney T, Singh A, et al. (2012) Painful arthritis and extremity rash in an 8-year-old boy. *Clin Infect Dis* 54: 1514–1515. https://doi.org/10.1093/cid/cir999
109. Addidle M, Pynn J, Grimwade K, et al. (2012) Epidural abscess caused by *Streptobacillus moniliformis*. *J Clin Microbiol* 50: 3122–3124. https://doi.org/10.1128/jcm.01004-12
110. McKee G, Pewarchuk J (2013) Rat-bite fever. *Cmaj* 185: 1346–1346. https://doi.org/10.1503/cmaj.121704

111. Flannery DD, Akinboyo I, Ty JM, et al. (2013) Septic arthritis and concern for osteomyelitis in a child with rat bite fever. *J Clin Microbiol* 51: 1987–1989. https://doi.org/10.1128/jcm.03139-12
112. Mackey JR, Melendez ELV, Farrell JJ, et al. (2014) Direct detection of indirect transmission of *Streptobacillus moniliformis* rat bite fever infection. *J Clin Microbiol* 52: 2259–2261. https://doi.org/10.1128/jcm.00259-14
113. Budair B, Goswami K, Dhukaram V (2014) Septic arthritis secondary to rat bite fever: a challenging diagnostic course. *BMJ Case Rep* 2014: bcr2014204086. https://doi.org/10.1136/bcr-2014-204086
114. Fenn DW, Ramoutar A, Jacob G, et al. (2014) An unusual tale of rat-bite fever endocarditis. *BMJ Case Rep* 2014: bcr2014204989. https://doi.org/10.1136/bcr-2014-204989
115. Adam JK, Varan AK, Pong AL, et al. (2014) Fatal Rat-Bite Fever in a child-San Diego county, California, 2013. *MMWR Morb Mortal Wkly Rep* 63: 1210.
116. Adizie T, Gayed M, Ravindran J (2014)Rat bite fever causing septic arthritis and osteomyelitis in a young man. *Rheumatology* 53: 57–57. https://doi.org/10.1093/rheumatology/keu096.001
117. Adams SH, Mahapatra R (2021) Rat bite fever with osteomyelitis and discitis: case report and literature review. *BMC Infect Dis* 21: 479. https://doi.org/10.1186/s12879-021-06172-x
118. Fansa AM, Tanios M, Ebraheim N (2014) A case of shoulder joint *Streptobacillus moniliformis* septic arthritis with severe subdeltoid bursitis. *Int Musculoskelet Med* 36: 117–119. https://doi.org/10.1179/1753615414Y.0000000031
119. Andany N, Showler A, Morris AM, et al. (2015) A 50-year-old woman with fever, rash, and polyarthritis. *Clin Infect Dis* 60: 1436–1437. https://doi.org/10.1093/cid/civ047
120. Ibrahim W, Gehani A, Eltayeb F (2015) Infective endocarditis after multiple rat bites in a patient with diabetic neuropathy: If not *Streptobacillus moniliformis*, what else should be suspected? *IJMBS* 7: 228–230. https://doi.org/10.4103/1947-489X.210290
121. Okamori S, Nakano M, Nakamura M, et al. (2015) A Japanese patient with a rare case of *Streptobacillus moniliformis* bacteremia. *J Infect Chemother* 21: 877–878. https://doi.org/10.1016/j.jiac.2015.08.003
122. Brown CM, Tsai G, Sanchez-Flores X (2015) Oh rats! Fever, rash and arthritis in a young woman. *BMJ Case Rep* 2015: bcr2015212240. https://doi.org/10.1136/bcr-2015-212240
123. Miraflor AP, Ghajar LD, Subramaniam S, et al. (2015) Rat-bite fever: an uncommon cause of fever and rash in a 9-year-old patient. *JAAD Case Rep* 1: 371–374. https://doi.org/10.1016/j.jdcr.2015.09.008
124. Akter R, Boland P, Daley P, et al. (2016) Rat bite fever resembling rheumatoid arthritis. Can *J Infect Dis Med Microbiol* 2016: 7. https://doi.org/10.1155/2016/7270413
125. Nei T, Sato A, Sonobe K, et al. (2015) *Streptobacillus moniliformis* bacteremia in a rheumatoid arthritis patient without a rat bite: a case report. *BMC Res Notes* 8: 1–5. https://doi.org/10.1186/s13104-015-1642-6
126. Sato R, Kuriyama A, Nasu M (2016) Rat-bite fever complicated by vertebral osteomyelitis: A case report. *J Infect Chemother* 22: 574–576. https://doi.org/10.1016/j.jiac.2016.01.023
127. Kawakami Y, Katayama T, Kishida M, et al. (2016) A case of *Streptobacillus moniliformis* infection with cutaneous leukocytoclastic vasculitis. *Acta Med Okayama* 70: 377–381. http://doi.org/10.18926/AMO/54596

128. Gill NK, Craft D, Crook T, et al. (2016) A teenager with sacroileitis, rash and fever caused by *Streptobacillus moniliformis* bacteremia. *Pediatr Infect Dis J* 35: 1364–1366. http://doi.org/10.1097/INF.0000000000001334
129. Kwon CW, Somers K, Scott G, et al. (2016) Rat bite fever presenting as palpable purpura. *JAMA Dermatol* 152: 723–724. https://doi.org/10.1001/jamadermatol.2015.6034
130. Nathan M (2017) A rare triple threat: Rat bite fever, endocarditis of ventricular septal defect patch, and multivalve endocarditis with sinus of valsalva fistula. *J Thorac Cardiovasc Surg* 153: 47–48. https://doi.org/10.1016/j.jtcvs.2016.11.012
131. Wegner AM, Look N, Haus BM (2017) Surgical management of multijoint septic arthritis due to rat-bite fever in a pediatric patient: a case study. *J Orthop Case Rep* 2017. https://doi.org/10.1155/2017/2183941
132. Eisenberg T, Poignant S, Jouan Y, et al. (2017) Acute tetraplegia caused by rat bite fever in snake keeper and transmission of *Streptobacillus moniliformis*. *Emerg Infect Dis* 23: 719. https://doi.org/10.3201/eid2304.161987
133. Suzuki K, Hirai Y, Morita F, et al. *Streptobacillus moniliformis* bacteremia in a pet shop employee: Case report and literature review (2017) *Open Forum Infect Dis* 4: ofx038. https://doi.org/10.1093/ofid/ofx038
134. Hammer A, Wolff D, Geißdörfer W, et al. (2017) A spinal epidural abscess due to *Streptobacillus moniliformis* infection following a rat bite: case report. *J Neurosurg* 27: 92–96. https://doi.org/10.3171/2016.12.SPINE161042
135. Scott J, Clarke C, Marchell R (2017) Petechiae, purpura, and hemorrhagic vesicles. *JAMA Dermatol* 153: 707–708. https://doi.org/10.1001/jamadermatol.2016.5965
136. Costa-Pinto J, Morley C, Hauser S (2017) A case of rat bite fever in a 12-year-old boy. *J Paediatr Child Health* 53: 84–86. https://doi.org/10.1111/jpc.13320
137. Kämmerer T, Lesmeister T, Wollenberg A, et al. (2021) Rat bite fever, a diagnostic challenge: case report and review of 29 cases. *J Dtsch Dermatol Ges* 19: 1283–1287. https://doi.org/10.1111/ddg.14526
138. Torres-Miranda D, Moshgriz M, Siegel M (2018) *Streptobacillus moniliformis* mitral valve endocarditis and septic arthritis: the challenges of diagnosing rat-bite fever endocarditis. *Infect Dis Rep* 10: 7731. https://doi.org/10.4081/idr.2018.7731
139. Ioffe V, Amir G, Zalzstein E, et al. (2018) *Streptobacillus moniliformis* endocarditis: an unusual case of pulmonary valve erosion resulting in free pulmonary regurgitation and aneurysm. *World J Pediatr Congenit Heart Surg* 9: 467–469. https://doi.org/10.1177/2150135116637808
140. Kasuga K, Sako M, Kasai S, et al. (2018) Rat bite fever caused by *Streptobacillus moniliformis* in a cirrhotic patient initially presenting with various systemic features resembling Henoch-Schönlein Purpura. *Intern Med* 57: 2585–2590. https://doi.org/10.2169/internalmedicine.9856-17
141. Abusalameh M, Mahankali-Rao P, Earl S (2018) Discitis caused by rat bite fever in a rheumatoid arthritis patient on tocilizumab-first ever case. *Rheumatology* 57: 1118–1120. https://doi.org/10.1093/rheumatology/key046
142. Nelson C, Rawling RA, Granato PA (2018) *Streptobacillus moniliformis* native valve endocarditis. *Clin Microbiol Newsl* 40: 74–76. https://doi.org/10.1016/j.clinmicnews.2017.11.002

143. Zhang WW, Hu YB, He GX, et al. (2019) Rat bite fever caused by *Streptobacillus moniliformis* infection in a Chinese patient. *BMC Infect Dis* 19: 1–5. https://doi.org/10.1186/s12879-019-4281-z
144. Rodino KG, Miller NE, Pethan KD, et al. (2019) The brief case: rat bite fever from a kiss. *J Clin Microbiol* 58: e00677-19. https://doi.org/10.1128/jcm.00677-19
145. Gaston DC, Peaper DR, Advani SD (2019) Fever, rash, and migratory polyarthralgias. *JAMA* 321: 1930–1931. https://doi.org/10.1001/jama.2019.4799
146. Khakwani A, Yusuf A (2019) E031 infectious vasculitis: a great mimic. *Rheumatology* 58: kez110. 030. https://doi.org/10.1093/rheumatology/kez110.030
147. Pena E, Jordão S, Simões MJ, et al. (2019) A rare cause of vertebral osteomyelitis: the first case report of rat-bite fever in Portugal. *Rev Soc Bras Med Trop* 53: e20190328. https://doi.org/10.1590/0037-8682-0328-2019
148. Giorgiutti S, Lefebvre N (2019) Rat bite fever. *N Engl J Med* 381: 1762. https://doi.org/10.1056/nejmicm1905921
149. Winther M, Jensen HS, Tarpgaard IH, et al. (2020) Case report: A fatal case of aortic and mitral valve endocarditis caused by *Streptobacillus moniliformis*. *Eur Heart J* 4: 1–6. https://doi.org/10.1093/ehjcr/ytaa254
150. Mulkareddy V, Bhalla V, Chhabria M, et al. (2020) A bite of a heart: a case of rat bite endocarditis. *Am J Respir Crit Care Med* 201: A3476. https://doi.org/10.1164/ajrccm conference.2020.201.1_MeetingAbstracts.A3476
151. Shadrin IY, Albitar HAH, Paim AC, et al. (2020) Migratory polyarthralgias and skin rash: Rat bite fever with a positive anti-cyclic citrullinated peptide. *MCP:IQ&O* 4: 223–227. https://doi.org/10.1016/j.mayocpiqo.2019.11.004
152. Pannetier LWX, Lombard E (2020) Rat bite fever in senior health medicine. *BMJ Case Rep* 13: e233451. https://doi.org/10.1136/bcr-2019-233451
153. Smallbones M, Monem M, Baganeanu M, et al. (2020) Near-fatal periprosthetic infection with *Streptobacillus moniliformis*: case and review. *J Bone Jt Infect* 5: 50–53. https://doi.org/10.7150/jbji.40635
154. Rai D, Tahir MW, Mulkareddy V, et al. (2020) Rat bite endocarditis: an unusual presentation. *J Am Coll Cardiol* 75: 2974–2974.
155. Khatib MY, Elshafei MS, Mutkule DP, et al. (2020) Rat bite fever: The first case report from Qatar. *Am J Case Rep* 21: e925647–925641. https://doi.org/10.12659/AJCR.925647
156. Onodera H, Uekita H, Watanabe T, et al. (2020) Rat-bite fever due to *Streptobacillus moniliformis* in a patient without bite history: an unexpected cause of consciousness disturbance. *Jpn J Infect Dis* 73: 85–87. https://doi.org/10.7883/yoken.JJID.2019.271
157. Crofton KR, Ye J, Lesho EP (2020) Severe recurrent *Streptobacillus moniliformis* endocarditis in a pregnant woman, and review of the literature. *Antimicrob Resist Infect Control* 9: 1–4. https://doi.org/10.1186/s13756-020-00789-4
158. Skarentzos Sr K, Papadopulos N, Deftereos SP, et al. (2021) Serious rodent bites to an 8-month-old infant due to child neglect. *Cureus* 13: e18493. https://doi.org/10.7759/cureus.18493
159. Wang NY, Osowicki J (2021) Rat-bite fever in a child without a bite. *Arch Dis Child* 106: 652–652. https://doi.org/10.1136/archdischild-2020-320327

160. Matthews C, Ausman S (2021) Rat-bite fever: Taking the bite out of a textbook case of *Streptobacillus moniliformis* blood stream infection. *IDCases* 25: e01199. https://doi.org/10.1016/j.idcr.2021.e01199
161. Roussel-Simonin C, Jousset AB, Knafo S, et al. (2021) *Streptobacillus moniliformis* subdural empyema in a homeless patient. *Clini Infect Pract* 12: 100098. https://doi.org/10.1016/j.clinpr.2021.100098
162. Uddin A, Phan T, Yassin M (2021) Septic polyarthritis caused by *Streptobacillus moniliformis*. *Emerg Infect Dis* 27: 3198. https://doi.org/10.3201/eid2712.210649
163. Yang FA, Li TJ, Ho MW, et al. (2021) Rat-bite fever with bacteremia and lower limb abscess formation caused by *Streptobacillus moniliformis*. *J Microbiol Immunol Infect* 55: 175–176. https://doi.org/10.1016/j.jmii.2021.10.003
164. Huang J, Shan X, Wang L, et al. (2022) *Streptobacillus moniliformis* bacteremia in a patient with reactive arthritis without a bite: a case report. *Jundishapur J Microbiol* 15: e126419. https://doi.org/10.5812/jjm-126419
165. Swan CD, Koirala A, Samarasekara H (2022) *Streptobacillus moniliformis* bacteraemia and septic arthritis in a child. *J Paediatr Child Health* 58: 1465–1467. https://doi.org/10.1111/jpc.15855
166. Bougioukas L, Vicks E, Hale AJ, et al. (2022) Rat bite fever in a patient with human immunodeficiency virus. *IDCases* 29: e01526. https://doi.org/10.1016/j.idcr.2022.e01526
167. Fokkema AT, Kampschreur LM, Pirii LE, et al. (2022) Rat bite fever in a total knee arthroplasty: an unusual case of periprosthetic joint infection. *Arthroplasty* 4: 1–4. https://doi.org/10.1186/s42836-022-00114-x
168. JM O'Brien CP, D Ridley (2022) Rat bite fever: a surreptitious cause of infective endocarditis. *J Am Coll Cardiol* 79.
169. Wallemacq S, Hing M, Mahadeb B, et al. (2023) *Streptobacillus moniliformis* right hand abscess and monoarthritis following a rat bite. *IDCases* 31: e01663.
170. Mohamed N, Albahra S, Haley C (2023) Rat-bite fever in a 34-year-old female. *Cureus* 15: e42453. https://doi.org/10.7759/cureus.42453
171. Błaż A, Zalewski J, Masiak A, et al. (2023) Rat bite fever mimicking ANCA-associated vasculitis. *Rheumatol Int* 43: 1957–1964. https://doi.org/10.1007/s00296-023-05369-4
172. Jones MK, Pedraza LC, Mann SC (2023) Acute polyarthritis with a finger wound: rat bite fever from *Streptobacillus moniliformis*. *Ann Intern Med* 2: e230198. https://doi.org/10.7326/aimcc.2023.0198
173. Fukushima K, Yanagisawa N, Imaoka K, et al. (2018) Rat-bite fever due to *Streptobacillus notomytis* isolated from a human specimen. *J Infect Chemother* 24: 302–304. https://doi.org/10.1016/j.jiac.2017.10.018
174. Ogawa Y, Kasahara K, Lee ST, et al. (2018) Rat-bite fever in human with *Streptobacillus notomytis* infection, Japan. *Emerg Infect Dis* 24: 1377. https://doi.org/10.3201/eid2407.171580
175. Kusuda T, Ryoko A, Yoshishige M, et al. (2020) Erosive polyarthritis caused by sepsis due to a novel species of *Streptobacillus notomytis*. *Mod Rheumatol Case* 4: 95–98. https://doi.org/10.1080/24725625.2019.1655246

176. Pongsuttiyakorn S, Kamolvit W, Limsrivanichakorn S, et al. (2021) Rat bite fever due to *Streptobacillus notomytis* complicated by meningitis and spondylodiscitis: a case report. *BMC Infect Dis* 21: 1–5. https://doi.org/10.1186/s12879-021-06715-2
177. Kawashima A, Kutsuna S, Shimomura A, et al. (2022) *Streptobacillus notomytis* bacteremia after exposure to rat feces. *Emerg Infect Dis* 28: 886. https://doi.org/10.3201/eid2804.204965
178. Matt U, Schmiedel J, Fawzy A, et al. (2021) Infection in a young immunocompetent male caused by *Streptobacillus felis*, a putative zoonotic microorganism transmitted by cats. *Clin Infect Dis* 72: 1826–1829. https://doi.org/10.1093/cid/ciaa968

AIMS Press

© 2024 the Author(s), licensee AIMS Press. This is an open access article distributed under the terms of the Creative Commons Attribution License (http://creativecommons.org/licenses/by/4.0)
